# LINE-1 ORF1p does not determine substrate preference for human/orangutan SVA and gibbon LAVA

**DOI:** 10.1186/s13100-020-00222-y

**Published:** 2020-07-11

**Authors:** Annette Damert

**Affiliations:** Primate Genetics Laboratory, German Primate Center, Leibniz Institute for Primate Research, Göttingen, Germany

## Abstract

**Background:**

Non-autonomous VNTR (Variable Number of Tandem Repeats) composite retrotransposons – SVA (SINE-R-VNTR-*Alu*) and LAVA (L1-*Alu*-VNTR-*Alu*) – are specific to hominoid primates. SVA expanded in great apes, LAVA in gibbon. Both SVA and LAVA have been shown to be mobilized by the autonomous LINE-1 (L1)-encoded protein machinery in a cell-based assay in *trans*. The efficiency of human SVA retrotransposition in vitro has, however, been considerably lower than would be expected based on recent pedigree-based in vivo estimates. The VNTR composite elements across hominoids – gibbon LAVA, orangutan SVA_A descendants and hominine SVA_D descendants – display characteristic structures of the 5′ *Alu*-like domain and the VNTR. Different partner L1 subfamilies are currently active in each of the lineages. The possibility that the lineage-specific types of VNTR composites evolved in response to evolutionary changes in their autonomous partners, particularly in the nucleic acid binding L1 ORF1-encoded protein, has not been addressed.

**Results:**

Here I report the identification and functional characterization of a highly active human SVA element using an improved *mneo* retrotransposition reporter cassette. The modified cassette (*mneoM*) minimizes splicing between the VNTR of human SVAs and the neomycin phosphotransferase stop codon. SVA deletion analysis provides evidence that key elements determining its mobilization efficiency reside in the VNTR and 5′ hexameric repeats. Simultaneous removal of the 5′ hexameric repeats and part of the VNTR has an additive negative effect on mobilization rates. Taking advantage of the modified reporter cassette that facilitates robust cross-species comparison of SVA/LAVA retrotransposition, I show that the ORF1-encoded proteins of the L1 subfamilies currently active in gibbon, orangutan and human do not display substrate preference for gibbon LAVA versus orangutan SVA versus human SVA. Finally, I demonstrate that an orangutan-derived ORF1p supports only limited retrotransposition of SVA/LAVA in *trans*, despite being fully functional in L1 mobilization in *cis*.

**Conclusions:**

Overall, the analysis confirms SVA as a highly active human retrotransposon and preferred substrate of the L1-encoded protein machinery. Based on the results obtained in human cells coevolution of L1 ORF1p and VNTR composites does not appear very likely. The changes in orangutan L1 ORF1p that markedly reduce its mobilization capacity in *trans* might explain the different SVA insertion rates in the orangutan and hominine lineages, respectively.

## Background

**Fig. 1:**
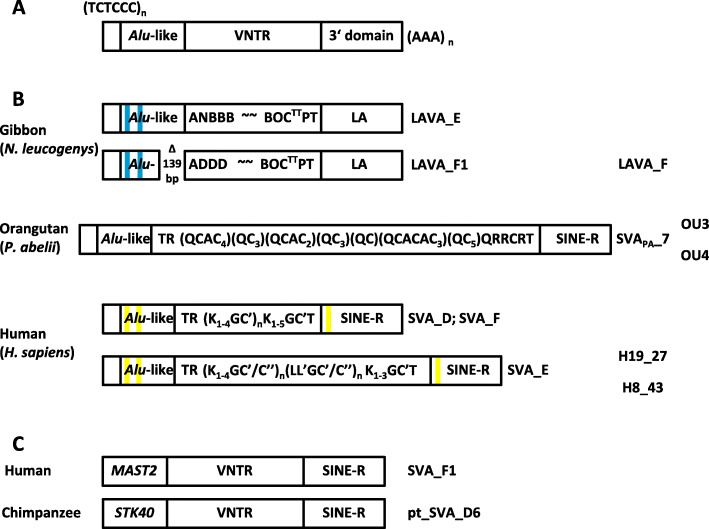
General structure of VNTR composite retrotransposons and SVA/LAVA subfamilies in hominoid primates. **a** Structure of SVA/LAVA. The elements are composed of (from 5′) hexameric repeats (TCTCCC)_n_, an *Alu*-like region, variable number of 36-49 bp tandem repeats (VNTR) and either a retrovirus-derived SINE (SINE-R in SVA) or a 3′ domain containing L1 and *Alu* fragments (LA in LAVA). They terminate with a poly A tail (AAA)_n_. **b** Currently active LAVA and SVA subfamilies in hominoid primates. Blue and yellow bars indicate short deletions relative to the ancestral SVA_A sequence. Tildes represent the apparently unstructured central part of gibbon LAVA. The VNTR subunit code is that described in Lupan et al. [4]. TR represents the invariable tandem repeats at the VNTR 5′ end. Note that the type and sequence of subunits in this part is not identical among subfamilies (for details see [4]). The overall structure of SVA_D elements in gorilla and chimpanzee corresponds to that shown for humans. LAVA_F, OU3, OU4, H19_27 and H8_43 denote the LAVA/SVA elements used in the study. The position indicates their subfamily affiliation. **c** Non-canonical SVAs in human and chimpanzee. In SVA_F1 and pt_SVA_D6 the hexameric repeat and larger part of the *Alu*-like regions are replaced by the first exons of *MAST2* and *STK40*, respectively

The mobile element landscape of hominoid primates (gibbon, orangutan, gorilla, chimpanzee and human) is characterized by the expansion of non-autonomous composite non-LTR (non-long terminal repeat) retrotransposons (SVA – SINE-R-VNTR-*Alu* [1, 2]; LAVA – L1-*Alu*-VNTR-*Alu* [3]) that are absent in Old World monkeys. SVA elements amplified in the hominids (orangutan, gorilla, chimpanzee and human); LAVA expanded in gibbon only. Figure 1a shows the structural organization of the elements: 5′ hexameric repeats (TCTCCC)_n_, a domain composed of two partial antisense *Alu* copies (*Alu*-like) and a region comprised of 36-50 bp variable number of tandem repeats (VNTR) are shared by SVA and LAVA. The 3′ end of SVAs (SINE-R – retrovirus-derived SINE) is derived from the endogenous retrovirus HERV-K; the LAVA 3′ end contains *Alu* and L1 fragments separated by simple repeats (Fig. 1b). Both SVA and LAVA evolve as hierarchical subfamilies [2, 5] displaying subfamily-specific nucleotide exchanges and small indels. However, by contrast to other non-LTR retrotransposons evolution of these composite elements does not only occur at the nucleotide level but also at the level of structural organization of the VNTR domain [4] (Fig. 1b).

The VNTR of gibbon LAVA elements is characterized by conserved subunit arrangements at both the 5′ and 3′ end of the domain. Orangutan SVAs are direct derivatives of the evolutionary oldest subfamily SVA_A. The VNTR of the evolutionary youngest orangutan subfamilies is composed of a fixed 5′ end (TR – tandem repeat) followed by arrays of Q and C subunits ((QCAC_4_)(QC_3_)(QCAC_2_)(QC_3_)(QC)(QCACAC_3_)(QC_5_)) and a fixed 3′ end. The phylogenetically most recent SVA elements in the hominines (SVA_D in gorilla and chimpanzee and SVA_D, SVA_E and SVA_F in human) display short deletions in both the *Alu*-like and SINE-R regions when compared to the ancestral SVA_A. In the VNTR a fixed 5′ part (TR) is followed by [(K_1-4_GC’)_n_] (SVA_D; SVA_F) or [(K_1-4_GC’/C″)_n_ (LL’GC’/C″)_n_] (SVA_E) variable length arrays. Overall, the hominine SVA VNTR is dominated by 49 bp G-rich K-type subunits whereas orangutan SVA VNTRs are enriched for short, 37 bp long C-type subunits [4].

In addition to the canonical SVAs depicted in Fig. 1b chimpanzee and human harbour non-canonical composite elements in which the 5′ hexameric repeats and the larger part of the *Alu*-like region are replaced by the first intron of *MAST2* (SVA_F1 in human [6–8]) and *STK40* (pt_SVA_D6 in chimpanzee [9]), respectively (Fig. 1c). Copy numbers of the composite non-LTR retrotransposons range from 1800 in gibbons (LAVA in *Nomascus leucogenys* [5]), 1800 (SVA in orangutan [10]) to 2800 (SVA in human [2]).

As non-autonomous elements VNTR-composite retrotransposons are dependent on the proteins encoded by the autonomous LINE-1 (L1) element for their mobilization [11–14]. Across hominoids SVA/LAVA “pair” with L1 partners belonging to different subfamilies: LAVA with L1PA4 in gibbons, SVA_pa_ with L1PA3 in orangutan and SVA_hs_ with L1PA1 in human. Given the requirement for L1-encoded proteins for VNTR-composite mobilization it can be hypothesized that LAVA and orangutan/human SVA evolved their specific structural features in response to the characteristics of the L1 subfamily active in the respective lineage. The primary interaction of RNAs to be retrotransposed by the L1 protein machinery occurs with the nucleic acid binding protein encoded by L1 ORF1 [15]. Mobilization of both SVA and LAVA is dependent on L1 ORF1p [12, 13]. Taken together these two facts suggest that the determinants for substrate preference of LINE1 subfamilies for LAVA versus orangutan SVA versus human SVA might reside in L1 ORF1p.

To date, three different human SVA elements and two LAVA elements have been characterized with regard to their capacity to be mobilized by L1-encoded proteins in *trans* in a cell-based assay [11–14, 16]. The retrotransposition rates reported for the human SVAs differ by three orders of magnitude from those observed for L1 in *cis* (4-5 × 10^− 5^ [11, 12] versus 1.3 × 10^− 2^ [17]). Recently published estimates for in vivo mobilization rates, however, show human SVA on par with human L1 (one in 63 births [18]). In addition, the relatively high number of disease-causing SVA insertions (16 [19–22], compared to 30 for L1 and 76 for *Alu* [19] with much higher copy number in the genome) points to a considerable activity of SVA elements in vivo. Taken together, the elements tested so far might not represent the currently active fraction of SVAs in the human genome. Orangutan SVAs have not been investigated in the cell-based assay. As a prerequisite for addressing the hypothesis of LAVA/SVA – L1 coevolution I report here the identification and functional characterization of a human SVA element considerably more active than those described previously. I also demonstrate that orangutan SVAs can be efficiently mobilized by human L1 in human cells. Finally, using codon-optimized L1 ORF1 chimeras, I show that LINE1 ORF1p derived from the three species under study does not determine substrate preference for gibbon LAVA versus orangutan SVA versus human SVA.

## Results

### Identification and isolation of potentially active human and orangutan SVA elements

Retrotransposition-competent SVA elements can be expected to lack potentially inactivating mutations/structural modifications (substitutions or indels (*Alu*-like region and SINE-R)/changes in the VNTR substructure relative to the subfamily consensus). There is a high probability for such elements to be found among source elements of recently integrated copies still displaying presence/absence polymorphism and among these polymorphic elements themselves.

In case of human polymorphic elements of the evolutionary youngest subfamilies SVA_E and SVA_F were extracted using dbRIP [23]. Detailed analysis of all full-length elements in the dataset identified a small group of SVA_E elements carrying a specific 6 bp insertion in the SINE-R region (SVA_E1; Additional file 1). The entire group comprises nine 5′ full-length elements; six out of them are polymorphic according to dbRIP. Based on analysis of the VNTR structure (Additional file 1) and on similarity to the group consensus sequence two elements (chr7:1,185,116-1,187,654 and chr8:43,033,761-43,036,378; hg19) were selected for amplification. One of them (chr8) displays an 11 bp deletion in the 3′ part of the SINE-R. The chr7 element was absent in all three human genomic DNAs tested. The chr8 element (H8_43) was amplified, sub-cloned and sequenced. The amplified sequence is provided in Additional file 2: Figure S1.

In orangutan the search was based on a previous analysis [4]. Unfortunately, the quality of the genome build (ponAbe2) available at that time permitted the identification of only very few 5′ full-length elements belonging to the evolutionary younger subfamilies SVA_PA__7–11. The full-length elements were genotyped in silico on available short read archives and most of them were found to be polymorphic. Three elements (all belonging to subfamily SVA_PA__7) were then amplified from genomic DNA of eight individuals (7x *Pongo abelii; 1x Pongo pygmaeus*). As expected, all of them were found to be polymorphic among the individuals tested (Additional file 1). Finally, the SVA containing alleles were sub-cloned and sequenced. The amplified sequences are provided in Additional file 2: Figures S2 and S3.

### A modified reporter cassette permits robust comparison of SVA mobilization rates across species

**Fig. 2:**
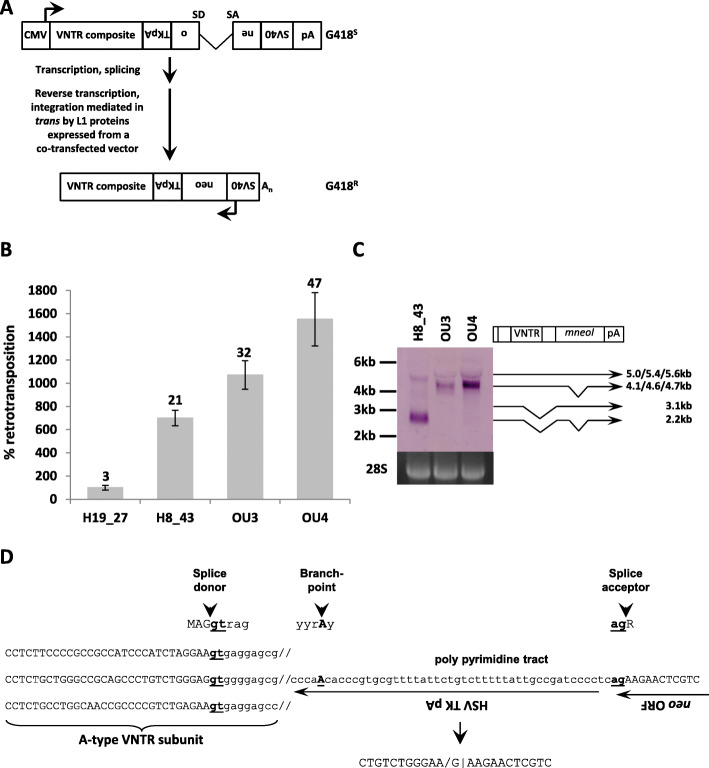
Human SVAs are spliced in the context of the *mneoI* reporter cassette. **a** Schematic representation of the cell-based retrotransposition assay. The element of interest is tagged with a reporter cassette containing a neomycin phosphotransferase (neo) coding region driven by the SV40 promoter and polyadenylated at an HSV TK poly A site in antisense. The neo open reading frame is interrupted by an intron in sense direction. Following transcription of the VNTR composite from the 5′ CMV promoter, the intron is spliced out and the RNA is polyadenylated at the downstream SV40 pA site. Mediated by the L1 proteins encoded on a cotransfected vector the RNA is then reverse transcribed and the cDNA copy inserted into the genome. A functional neomycin phosphotransferase can now be generated from the uninterrupted coding region – giving rise to G418 resistant (G418^R^) cells once retrotransposition has occurred. SD – splice donor; SA – splice acceptor; G418^S^ – G418 sensitive (**b**) Retrotransposition assay of *mneoI*-tagged human (H19_27, H8_43) and orangutan (OU3, OU4) SVA elements. Retrotransposition rates +/− SEM are shown relative to H19_27 (100%). Average colony counts are given on top of each column. *n* ≥ 3 (**c**) Northern blot analysis of *mneoI*-tagged SVA transcripts. In case of the human SVA (H8_43) splicing between the VNTR and the *mneoI* cassette generates additional mature RNAs schematically depicted on the right. Lengths are given in the order of loading on the gel. **d** Structure of the H8_43 VNTR-neo splice variants as determined by RT-PCR. Nucleotides important for splicing are bold and underlined; intron sequence is in lowercase

The human (H8_43) and two of the orangutan (OU3 – chr19:59,431,118-59,434,697 and OU4 – chr1:218,026,414-218,030,602; ponAbe2) elements were subsequently tested in a cell-based retrotransposition assay using the *mneoI* reporter cassette [24] (in pCEPneo [12]) and L1RP (pJM101/L1RP∆neo [17]) as driver in Hela HA cells. Figure 2a shows the principle of the assay. A previously characterized human SVA_E element (H19_27 in pAD3/SVA_E [12]) was also included in the experiments. As shown in Fig. 2b, the two orangutan elements were found to be 10-15x more active than H19_27. The newly identified human H8_43 was mobilized seven times more efficiently than H19_27. The high retrotransposition rates observed for the orangutan SVAs were surprising against the background that they contain the “ancestral” SVA_A-type *Alu*-like region also present in gibbon PVA (*PTGR*-VNTR-*Alu*) and FVA (FRAM-VNTR-*Alu*) elements. Their *Alu*-like domains had been shown to dramatically decrease the mobilization rate when fused to the VNTR and SINE-R of the human H19_27 SVA_E element [13]. Northern blot analysis (Fig. 2c) revealed that the H8_43_*mneoI* transcript is extensively spliced; the correctly spliced variant (γ-globin intron only) is barely detectable. In case of the two orangutan elements only the *mneoI*-single spliced transcripts are detected. Considering the obvious differences in the processing of *mneoI*-tagged human and orangutan SVAs I concluded that a robust cross-species comparison of SVA mobilization rates is not possible using the established *mneoI* reporter cassette.

**Fig. 3:**
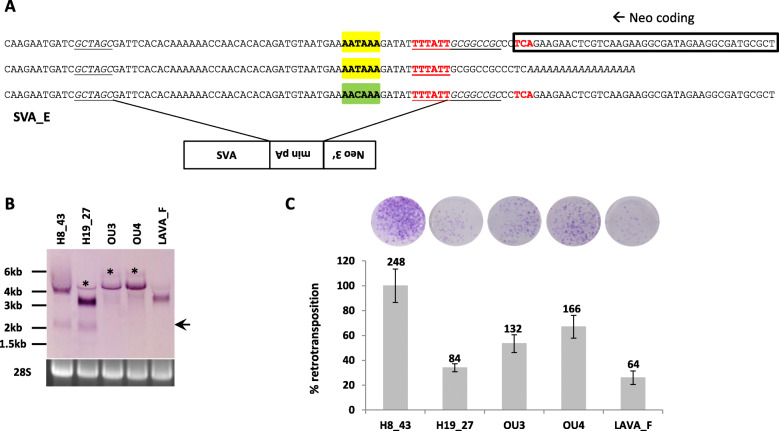
A modified reporter cassette – *mneoM* – minimizes splicing of human SVAs. **a** Sequence of the modified *mneoM* reporter cassette. The HSV TK polyA of *mneoI* is replaced by a minimal functional polyA signal (min pA). The resulting polyA signal leading to premature termination upstream of the reporter cassette (middle line, yellow box) is changed to AACAAA (boxed in green). The polyA signal facilitating 3′ end formation of the neomycin phosphotransferase (Neo) RNA is in red and underlined. Restriction sites used for cloning are italicized and underlined. **b** Northern blot analysis of *mneoM*-tagged human and orangutan SVA and LAVA RNAs. Expected sizes of the transcripts spliced in the reporter cassette only are: H8_43–4.1 kb; H19_27–3.3 kb; OU3–4.6 kb; OU4–4.7 kb; LAVA_F – 3.6 kb. Asterisks indicate unspliced transcripts; the arrow marks double-spliced mRNAs of the *mneoM*-tagged human SVAs. **c** Retrotransposition of *mneoM*-tagged human and orangutan SVAs and a gibbon LAVA element. Retrotransposition rates +/− SEM are shown relative to H8_43 (100%). H8_43; H19_27 – human SVA_E; OU3; OU4 – orangutan SVAs. Average colony counts are given on top of each column. *n* = 4

RT-PCR of the human SVA-*mneoI* splice variants established that the polypyrimidine tract and branchpoint at the acceptor site are provided by the *mneoI* HSV TK pA region (Fig. 2d [13];). I, therefore, decided to replace this part of the cassette by a minimal functional polyadenylation signal [25]. To prevent premature polyadenylation upstream of the reporter cassette the antisense polyA signal in the fragment was modified (Fig. 3a; for details on functional validation see Additional file 2, Fig. S4). Subsequently, all available SVA sequences (H19_27/SVA_E, H8_43/SVA_E, OU3 and OU4) as well as the previously characterized gibbon LAVA_F element [13] were combined with the modified reporter cassette named *mneoM* (modified *mneo*).

Northern blot analysis following transfection into Hela HA cells (Fig. 3b) revealed a considerable reduction in the amount of double-spliced (VNTR-neo stop and *mneoM*-intron) human SVA transcripts (arrow). Although splicing to the neo^R^ stop codon could not completely be abolished (only one of the three donor sites appears to be used according to RT-PCR analysis), the majority of the transcripts can now contribute to emergence of G418 resistant colonies in the cell-based retrotransposition assay.

Subsequent co-transfection of the constructs with pJM101/L1RP∆neo yielded retrotransposition rates > 1.9 × 10^− 3^ for the human H8_43/SVA_E element. Integration sites determined for three G418-resistant colonies show the hallmarks of L1-mediated retrotransposition: they are flanked by target site duplications (14-16 bp) and terminate with polyA tails of variable lengths (Additional file 2: Figure S5). The previously characterized human H19_27/SVA_E and LAVA_F elements were both mobilized at about 30% of H8_43. This is in contrast to published data using the *mneoI* cassette that demonstrated a twofold higher mobilization rate for the LAVA element when compared to H19_27 [13]. The two orangutan elements retrotransposed at 50–70% of the rate observed for H8_43/SVA_E (Fig. 3c). Overall, the results clearly show that splicing of human SVAs in the context of the established *mneoI* cassette confounds the results obtained in the cell-based retrotransposition assay.

### The VNTR and 5′ hexameric repeats determine mobilization capacity of human SVA

**Fig. 4:**
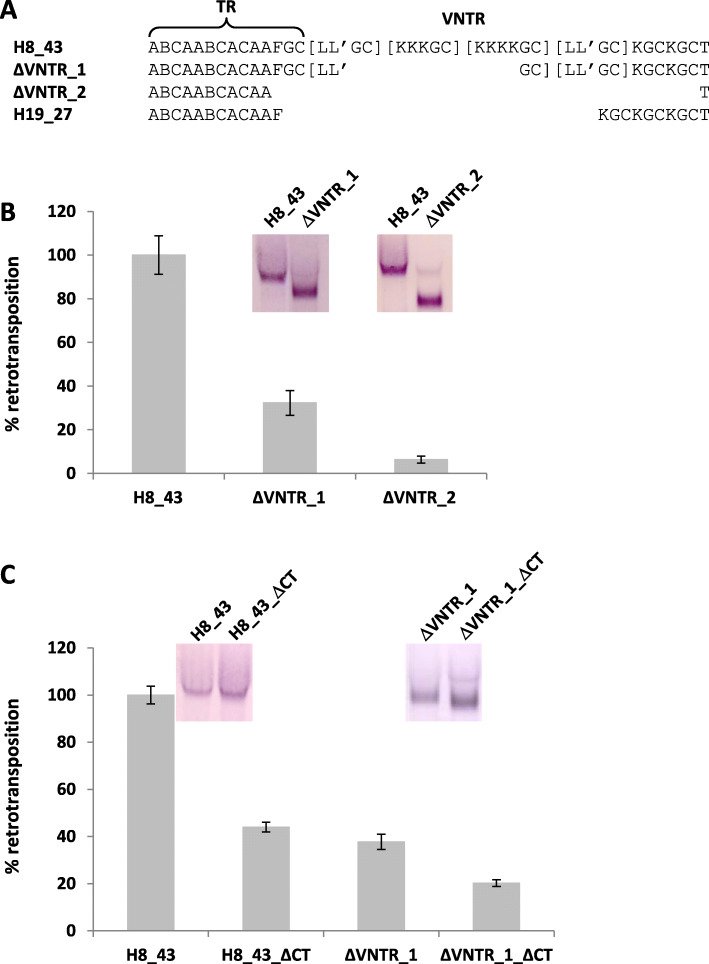
The VNTR and 5′ hexameric repeats determine mobilization capacity of human SVA. **a** VNTR structure of the H8_43 deletion mutants. VNTR subunits are encoded as in Lupan et al. [4]. Subunit arrays are bracketed. The VNTR subunit structure of H19_27 is given for comparison. TR – Tandem Repeat, fixed 5′ part of the domain; VNTR – variable length central part of the domain. **b** „In-frame“-deletions in the VNTR reduce SVA mobilization rates up to 90%. **c** Deletion of both the central part of the VNTR and the 5′ hexameric repeats has an additive effect. Retrotransposition rates +/− SEM are shown relative to the full-length element (100%). *n* = 3 for each independent set of experiments

A previous study has identified the 5′ hexameric repeat/*Alu*-like region as the “minimal active human SVA retrotransposon” [16]. The importance of this domain has also been supported by other reports employing deletion analysis [12] or domain swaps [13]. Deletion of the 5′ hexameric repeats alone has been shown to reduce retrotransposition rates by 75% [16]. Results obtained with regard to the function of the VNTR have been contradictory: larger deletions led to decrease in mobilization, whereas a shorter deletion resulted in an increase in the retrotransposition rate [16]. Here, “VNTR-slippage-mutants” generated in the course of re-amplification of the SVA elements by the thermostable polymerase offered the unique opportunity to study the effect of removal of parts of the VNTR in a setting comparable to the situation in vivo where slippage of the replication polymerase is the most likely mechanism producing changes in VNTR length and structure [4]. One of the deletion mutants tested (∆VNTR1) lacks the two central {K_n_GC} arrays; in the other one (∆VNTR2) the 3′ part of the fixed TR part and the entire variable part has been lost through slippage (Fig. 4a). In the cell-based retrotransposition assay ∆VNTR1 is mobilized at around 30% the level of the full-length element (similar to the level of H19_27 with a comparable VNTR length – cf. Figure 3c); ∆VNTR2 reaches only about 5%. As evidenced by Northern blotting the reduction in the mobilization rates cannot be attributed to a decrease in the steady-state level of the RNAs (Fig. 4b). In case of one of the orangutan elements (OU3), deletion of the VNTR (fusion of the 5′ and 3′ terminal repeat subunits) completely abolished retrotransposition (not shown).

A further set of experiments was designed to establish the function of the 5′ hexameric repeats in the context of the newly identified active SVA_E element and its possible interplay with the VNTR. As shown in Fig. 4c, deletion of the hexamers led to a 60% decrease in the mobilization rate. Combination of the hexamer and VNTR1 deletions reduced retrotransposition rates by 80%. In neither case the RNA steady state level has been affected. Taken together, these results suggest that the two domains might act cooperatively to define mobilization capacity.

### L1 ORF1p does not determine substrate preference for gibbon LAVA versus orangutan SVA versus human SVA

Ideally, substrate preference of species-specific L1 should be tested using multiple elements derived from that species. A pilot study using genomic copies of gibbon and orangutan L1 elements, however, failed.

**Fig. 5:**
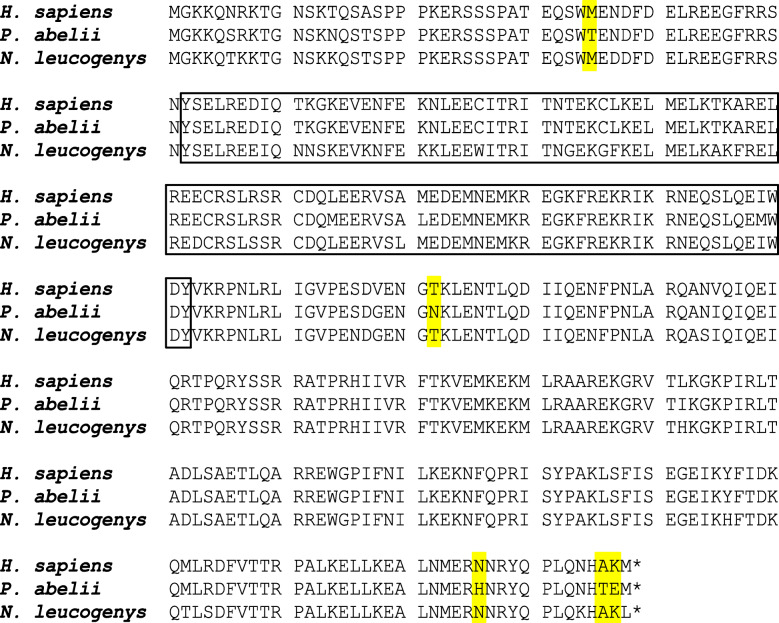
L1 ORF1p sequences tested for retrotranspositional activity in *cis* and *trans*. Substitutions (outside the coiled-coil domain) specific to orangutan ORF1p are highlighted in yellow. The coiled-coil domain is boxed

**Fig. 6:**
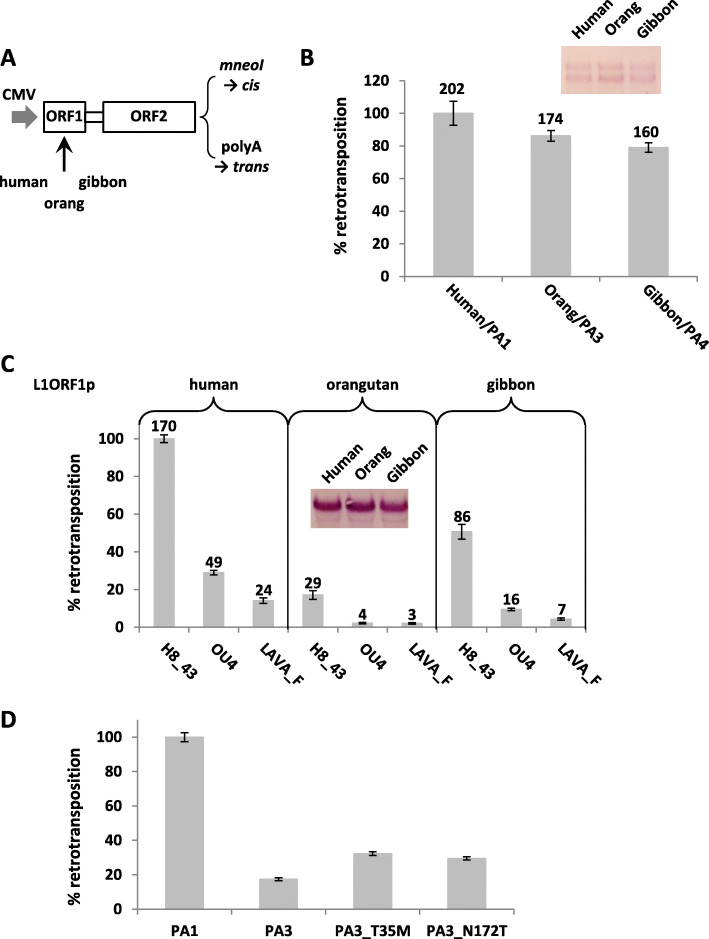
L1 ORF1p does not confer substrate preference for human SVA versus orangutan SVA versus gibbon LAVA. **a** Schematic representation of the L1 constructs used. Expression of codon-optimized species-specific ORF1 and human (L1PA1) ORF2 is driven by a CMV promoter. Reporter constructs used to assay for L1 mobilization in *cis* carry the *mneoI* reporter cassette; expression constructs for SVA/LAVA mobilization in *trans* a polyadenylation signal directly following ORF2. **b** Mobilization of the L1 ORF1p chimeras in *cis*. Retrotransposition rates +/− SEM are shown relative to the human (L1PA1) construct (100%). Average colony counts are given on top of each column (*n* = 4). **c** Mobilization of human and orangutan SVAs and gibbon LAVA in *trans*. Retrotransposition rates +/− SEM are shown relative to the combination of human L1 – human SVA (100%). Average colony counts are given on top of each column (*n* = 6). (**d**) Mutation of PA3 ORF1p amino acids 35 and 172 does not rescue mobilization capacity for SVA in *trans*. L1PA1, the chimeric L1PA3-PA1 and two mutants thereof were tested in combination with the *mneoM*-tagged human H8_43 SVA element. Retrotransposition rates +/− SEM are shown relative to the human (L1PA1) construct (100%, *n* = 4). The insets show Northern blot analyses of the RNAs transcribed from the respective L1 reporter (**b**) or expression (**c**) constructs

Mobilization of both SVA and LAVA is dependent on L1 ORF1p [12, 13]. To address a possible intra-species preference of L1 subfamily ORF1-encoded proteins for SVA/LAVA, I generated chimeras containing codon-optimized ORF1 sequences corresponding to the currently active subgroups (consensus sequences) of L1PA4 (gibbon) and L1PA3 (orangutan) and an established inter-ORF and codon-optimized ORF2 available in pBS-L1PA1-CH-mneo [26]. Codon optimization for mouse and human L1 elements has been shown to result in improved transcription, increased protein expression and mobilization rates in cell-based retrotransposition assays [26–28]. The protein sequences of the ORF1-encoded proteins are shown in Fig. 5, the general organization of the constructs used in Fig. 6a. The chimeras were first tested for retrotransposition in *cis*. As shown in Fig. 6b, there are no major differences to be observed. The codon-optimized L1PA1 and chimeric elements lacking the *mneoI* reporter cassette were then transferred into the episomal pCEP4 vector to assess their capacity to mobilize VNTR-composite elements in *trans* (Fig. 6c). For this assay the 11 bp deletion in the SINE-R region of the human SVA_E H8_43 was corrected to obtain an element corresponding to the subgroup consensus. The modification did not significantly affect mobilization rates when L1RP was used as the autonomous partner (not shown).

If there is L1 ORF1p-mediated substrate preference then the human element should be mobilized most efficiently by the human L1PA1; orangutan SVA by the L1PA3-PA1 chimera and gibbon LAVA by the L1PA4-PA1 chimera. This, however, was not found to be the case: the human SVA_E element is the most efficiently mobilized with all three ORF1-encoded proteins, followed by orangutan SVA and gibbon LAVA. The finding that the L1PA4-PA1 chimera shows only about 50% of the activity of L1PA1 is not really surprising given the phylogenetic distance between the two L1 subfamilies. However, the very low retrotransposition activity of the L1PA3-PA1 chimera in *trans* was completely unexpected given that the construct showed only slightly diminished mobilization capacity in *cis* when compared to L1PA1.

Outside the coiled-coiled domain mediating trimerization [29] two of the amino acid exchanges specific to orangutan ORF1p reside in the N-terminal region (T35) and central RRM (RNA recognition motif) domain (N172), respectively. Both domains have been characterized in human ORF1p with regard to their role in L1 mobilization in *cis* [30, 31], however, no specific function has been assigned to either of the residues in question (amino acids 35 and 172). In an attempt to identify amino acids exchanges that might be responsible for the reduced mobilization capacity of orangutan (PA3) ORF1p for SVA/LAVA in *trans*, I mutated the two residues to obtain the sequence present in human (PA1) and gibbon (PA4) ORF1p (T35M, N172T). Although an increase in human SVA H8_43 retrotransposition rates could be observed for both mutants, mobilization levels did not reach those obtained for the human ORF1p (Fig. 6d). Mobilization in *cis* has not been affected by the two mutations (not shown).

## Discussion

After *Alu* and L1, SVA/LAVA are the third largest group of non-LTR retrotransposons in hominoid primates [2]. They can act as insertional mutagens (for review see [32] and can co-mobilize sequences at both their 5′ [6, 7] and 3′ [33] ends. SVAs have also been shown to function as exon trap [7] and to be co-opted as regulatory sequence [34]. Despite this obvious impact on genome evolution and gene expression, their mechanism of mobilization and their amplification dynamics in evolution are not well understood.

Estimates based on a phylogenetic study (one in 916 births) pointed at a relatively low in vivo mobilization rate when compared to *Alu*, the other non-autonomous non-LTR retrotransposon in hominoid genomes (one in 21 births [35]). Results obtained in vitro in a cell-based retrotransposition assay appeared in agreement with these estimates: Hancks and colleagues reported an approximately 30-fold higher mobilization rate for *Alu* when compared to a (canonical) SVA element [11]. Against this background it has been disputed that SVA is indeed a preferred substrate of the L1-encoded proteins mediating its mobilization.

A recent pedigree-based analysis, however, resulted in a much higher estimate of SVA in vivo retrotransposition rates (one in 63 births) – comparable to that found for L1 (one in 63 births [18]) and in obvious contrast to the low rates observed in vitro. The results presented here now clearly show that SVA can be mobilized with high efficiency in cell culture. The elements previously characterized for their mobilization potential in vitro were identified based on (i) the ability to generate human-specific offspring (H2D – [16, 33]), (ii) the sequence similarity to the SVA_D consensus sequence (H11D – [16]) and (iii) the sequence identity to a reported disease-causing SVA insertion (SVA_E H19_27 –[12, 36]), respectively. The results presented here suggest that affiliation to a subgroup containing both polymorphic and fixed elements taken together with low divergence from the subgroup consensus (*Alu*-like region and SINE-R) and a VNTR structure corresponding to the subgroup “consensus” could be a suitable basis for the identification of potentially active SVA elements. Analysis of the entire human SVA_E subfamily identified five such subgroups. Details for two of them are provided in Additional file 3.

The results also show that the comparatively low in vitro mobilization rates reported previously can, to a large extent, be attributed to an experimental artefact: splicing of the SVA VNTR to the reporter cassette results in mature transcripts that cannot contribute to the fraction of G418 resistant cells following retrotransposition because they lack the stop codon and polyadenylation signal of the neomycin phosphotransferase. Possibly, the large amounts of double-spliced RNA produced also reduce the overall visible/detectable retrotransposition rate by acting as a “dominant-negative”: the 5′ hexameric repeat/ *Alu*-like region that constitutes the “minimal active human SVA retrotransposon” [16] and presumably mediates preferred interaction of SVAs with the L1-encoded proteins is present in the double-spliced RNA.

With regard to SVA functional domains the results obtained provide further support for the importance of the 5′ hexameric repeats in L1-mediated mobilization. Deletion of the domain leads to a decrease of 60% in the retrotransposition rate. Hancks et al. reported a 75% reduction in the context of SVA element H2D [16]. However, the hexameric repeat region of human SVAs is heterogeneous in both sequence and length. In SVA_E elements the TCTCCC repeats are frequently interspersed with Gs at regular intervals (e.g. in the previously described SVA H19_27). Preliminary results suggest that indeed there may be differences between elements with regard to the contribution of the 5′ hexameric repeats to overall mobilization capacity.

Previous results concerning the role of the central VNTR yielded conflicting results. Whereas complete deletion negatively affected mobilization, partial deletion led to a more than 50% increase [16]. However, the deletion mutants investigated were generated using restriction enzyme digestion that does not (i) accurately remove arrays of VNTR subunits and (ii) leaves subunits at the 5′ end of the domain and deletes the 5′ most part of the SINE-R as well. Thus, the constructs do not precisely reflect VNTR shortening the way it most likely occurs through polymerase slippage in vivo [4]. Experiments performed here with “VNTR-slippage-mutants” now provide clear evidence that the VNTR is a major determinant for efficient mobilization of SVA elements in both human and orangutan. For LAVA – the VNTR-composite family expanding in gibbons – it has been shown that either the length or a particular, as yet undefined, VNTR structure mediate efficient mobilization [4]. Thus, the central repetitive domain appears to play a key role in the amplification process across VNTR-composite families in hominoid primates. For a robust conclusion, however, analysis of additional SVA and LAVA elements will be required.

From an evolutionary point of view VNTR shortening by polymerase slipping could be considered to represent an inbuilt inactivation mechanism. An interesting point to be addressed in the future would be how fast this process occurs compared to random mutation leading to loss of activity in *Alu* elements.

Based on the finding that only a small number of L1 subfamilies were amplified intensively during the burst of *Alu* and processed pseudogene formation 40–50 million years (myrs) ago, Ohshima et al. hypothesized that “proteins encoded by members of particular L1 subfamilies acquired an enhanced ability to recognize cytosolic RNAs in *trans*” [37]. A later experimental study, however, could not find any evidence for coevolution between *Alu* and L1 [38]. Whereas *Alu* subfamilies differ by nucleotide exchanges and small indels only, VNTR composite retrotransposons display more pronounced differences across hominoid primates. LAVA is the dominant family in gibbon; orangutan SVAs are direct descendants of the ancestral SVA_A as far as the *Alu*-like domain and SINE-R are concerned and currently active elements in the hominines derive from SVA_D with its specific deletions in the *Alu*-like region and SINE-R [2]. In addition, there are marked differences in the subunit structures of the VNTR between LAVA, orangutan SVA and hominine SVAs [4]. Thus, by contrast to *Alu*, coevolution of VNTR-composites and L1 at the lineage/species level appeared to be possible. Given the dependence of VNTR-composite retrotransposition on L1 ORF1p [12, 13], changes mediating preferential mobilization of one or the other type (LAVA – orangutan SVA – human SVA) by a particular L1 subfamily could be expected to reside in this protein. The results obtained for the SVA/LAVA elements tested here, however, do not support this hypothesis. Indifferent of the ORF1p encoded in the constructs the human SVA is the most efficiently mobilized element. A preferred interaction of the human element with host factors involved in retrotransposition in the human cell environment might be an explanation for this observation. It will be interesting to see whether the preference of ORF1p for a particular VNTR-composite family changes with the cellular context, e.g. in orangutan or gibbon cells. In addition, it would be desirable to corroborate the results obtained with the analysis of more SVA/LAVA elements – also against the background that the now available orangutan genome build (ponAbe3) permits the generation of more reliable “consensus” VNTR substructures (Additional file 4) and, consequently, a more specific selection of potentially active SVAs from a wider range of sequenced and correctly assembled 5′ full-length elements.

In the absence of coevolution with its autonomous partner L1 SVA/LAVA could also have evolved to evade host repression. Turelli et al. [39] noticed that the human-specific subfamilies SVA_E and SVA_F are “less frequently associated with TRIM28 (a KRAB-zinc finger protein (ZFP) cofactor involved in transcriptional repression) than their older counterparts” and reasoned that “this could be because not enough time elapsed since they invaded the genome for KRAB-ZFPs or other TRIM28-tethering proteins recognizing their sequence to have been selected”.

Given the failure of detecting *Alu*-L1 coevolution [38], the finding that L1 ORF1p does not confer substrate preference in human cells did not really come as a surprise. The greatly reduced *trans* mobilization activity of the PA3-PA1 chimera, however, did – in particular against the background that the ORF1p protein encoded appears to be fully functional in L1 retrotransposition in *cis*. The multiple alignment of the ORF1p sequences reveals five amino acid exchanges specific to the orangutan protein outside the coiled-coil domain required for trimerization (Fig. 5). Substitution of two of these residues (T35 and N172) did not rescue orangutan ORF1p mobilization capacity in *trans* (compared to human PA1). It remains to be seen whether exchange of the C-terminal divergent amino acids or a combination of mutations (possibly including the orangutan-specific residues in the coiled-coiled domain) “restores” activity. From another point of view the greatly reduced capacity of the orangutan protein to mediate mobilization in *trans* might explain the lower insertion rate of SVA in the orangutan lineage. Based on a number of 1800 SVA elements in the genome of *P. abelii* (all lineage-specific), the lineage-specific insertion rate per myrs would be ca. 120 (split-time from hominines 14–16 myrs ago [10];). By contrast, the human genome harbours 1395 species-specific SVAs [9] – resulting in a lineage-specific insertion rate of ca. 280 per myrs (split-time from chimpanzee 4–6 myrs ago). However, a direct comparison of these numbers might be misleading: to date there is no information available about the SVA expansion dynamics in orangutan over the last 14–16 myrs. An approximately constant rate over the entire period of time and bursts of amplification are equally possible. In addition, the lineage-specific evolution of SVA’s autonomous partner, L1, in the orangutan lineage will have to be taken into account.

## Conclusions

SVAs can be mobilized with high efficiency in tissue culture – they are indeed a preferred substrate of the L1-encoded proteins. Modification of the retrotransposition reporter cassette to minimize splicing of human SVA facilitates robust comparison of VNTR composite mobilization across species and provides an essential tool for the analysis of these elements. Results obtained on SVA functional domains confirm earlier data on the role of the 5′ hexameric repeats [16] and assign a critical function to the VNTR in accordance with published findings for LAVA [4].

The results obtained in human cells do not provide any evidence for co-evolution between L1 ORF1p and VNTR composite elements across hominoids, suggesting that host factors most likely were or are involved in shaping the interaction between the autonomous and non-autonomous partners – at the root of each of the lineages (Hylobatidae, Ponginae, Homininae) and/or in the cellular environment of the present day species.

## Methods

### Amplification and cloning of human and orangutan SVA elements

Elements were amplified from genomic DNA using primers in the flanking sequence and Phusion HSII (Thermo Scientific). Orangutan DNA was obtained from the Gene Bank of Primates at the German Primate Center. Primer sequences are provided in Additional file 2: Table S6. To facilitate melting of the VNTR, the denaturation time was extended to 30s and 3% DMSO was added to the reaction mix. Amplicons were subcloned into pJET 1.2 (Thermo Scientific) and sequenced. To obtain complete VNTR sequences, subclones containing the VNTR 5′ and 3′ ends, respectively, were generated using SmaI (H8_43) or MscI (OU3, OU4). 5′ primers localized directly upstream of the CT hexameric repeats and 3′ primers designed to exclude the elements’ polyadenylation signals were used for re-amplification. KpnI and NheI recognition sites, respectively, were introduced into the upstream and downstream re-amplification primers. Amplicons were again subcloned into pJET 1.2, sequenced and transferred into pCEP Neo [12] and pCEP_*mneoM* via KpnI/NheI. The human SVA H8_43 displays an 11 bp deletion in the SINE-R region when compared to SVA_E and to the subgroup consensus sequences. To obtain a plasmid with a consensus SVA_E SINE-R for cross-species comparison, the missing 11 bp were introduced by site-directed mutagenesis (NEB Q5 kit).

### Modification of the *mneoI* reporter cassette: pCEP_*mneoM*

The minimal polyA signal [25] was excised NotI/ClaI from pGL3basic (Promega) and subcloned into pBII (KS+) yielding pB_syn_pA. The 3′ end of the *mneoI* cassette (lacking the HSV TK pA signal) was amplified from pCEP Neo [12] using the primers Neo_STOP_Not 5′ GGCGGCCGCCCTCAGAAGAACTCGTC 3′ and mneo_Xho_REV 5′ CCTCGAGACTAAAGGCAAC 3′, subcloned into pJET 1.2 (Thermo Scientific), and subsequently cloned upstream of the minimal pA signal in pB_syn_pA via SacI/blunt-XbaI/blunt and NotI. The polyA signal present in the antisense orientation was then changed to AACAAA by site-directed mutagenesis using the NEB Q5 kit. The fragment containing the modified minimal pA signal, the 3′ part of the neo^R^ coding sequence and the 5′ part of the *mneoI* intron was then transferred to pCEP Neo NheI/blunt-ClaI/blunt and XhoI to replace the respective part of the original *mneoI* cassette.

### Retrotransposition reporter cassette-containing constructs

All SVA and LAVA elements were cloned KpnI/NheI upstream of the respective reporter cassette. Details on the construction of the human SVA_E H8_43 deletion mutants can be obtained from the author. L1PA chimeras were generated by exchanging ORF1 in pBS-L1PA1-CH-mneo [26] NheI/BsmBI with the respective gibbon or orangutan sequence obtained as synthesized and cloned fragments in pMA-RQ (Invitrogen).

#### Orangutan SVA

pAD14 – orangutan SVA OU3 in pCEPNeo (*mneoI* reporter cassette).

pAD15 – orangutan SVA OU4 in pCEPNeo (*mneoI* reporter cassette).

pAD29 – orangutan SVA OU3 in pCEP_*mneoM* (*mneoM* reporter cassette).

pAD30 – orangutan SVA OU4 in pCEP_*mneoM* (*mneoM* reporter cassette).

#### Human SVA

pAD3 [12] – human SVA_E H19_27 in pCEPNeo (*mneoI* reporter cassette).

pAD24 – human SVA_E H8_43 in pCEPNeo (*mneoI* reporter cassette).

pAD27 – human SVA_E H19_27 [12] in pCEP_*mneoM* (*mneoM* reporter cassette).

pAD25 – human SVA_E H8_43 in pCEP_*mneoM* (*mneoM* reporter cassette).

∆VNTR1 – human SVA H8_43 internal deletion of VNTR subunits 17–28.

∆VNTR2 – human SVA H8_43 internal deletion of VNTR subunits 12–49.

H8_43_∆CT – human SVA H8_43 lacking the 5′ terminal hexameric repeats.

∆VNTR1_∆CT – human SVA H8_43 lacking the 5′ terminal hexameric repeats and VNTR subunits 17–28.

pAD26 – human SVA_E H8_43 with corrected 11 bp deletion in pCEP_*mneoM* (*mneoM* reporter cassette).

#### Gibbon LAVA

pAD28 – gibbon LAVA_F [13] in pCEP_*mneoM* (*mneoM* reporter cassette).

#### L1PA constructs

pBS-L1PA1-CH-mneo was a gift from A. Roy-Engel (Addgene plasmid # 51288; http://n2t.net/addgene:51288; RRID:Addgene_51,288) [26].

pBS-L1PA3-PA1-CH-mneoI – orangutan L1 ORF1/human ORF2.

pBS-L1PA4-PA1-CH-mneoI – gibbon L1 ORF1/human ORF2.

### L1 expression vectors

pJM101 L1RP∆neo [17] – kindly provided by J. Moran.

The L1PA1 and the chimeric L1PA expression vectors were generated by transferring the respective elements from the pBS-L1PA1-CH-mneo vectors EcoRI/blunt/NheI into pCEP4 (BamHI/blunt/NheI).

pCEP_L1PA1.

pCEP_L1PA3-PA1.

pCEP_L1PA4-PA1.

### Tissue culture and retrotransposition assays

Hela HA cells (a gift from J. Moran) were cultured in DMEM (Gibco) 4.5 g/l Glucose, 10% FCS. Cell-based retrotransposition assays were carried out as described previously [12, 40]. Briefly, 1.5 × 10^5^ cells per well were seeded in 6-well plates. 24 h after seeding cells were transfected with 0.5 μg each of the L1 expression plasmid and the *mneoI*/*mneoM*-tagged reporter construct using X-tremeGENE 9 (Roche) according to the manufacturer’s instructions. G418 selection (Sigma; 400 μg/ml) was started 72 h after transfection and continued for 12 days. Resulting colonies were then stained with Giemsa and counted.

### RNA isolation and analysis

For RNA analysis Hela HA cells were transfected with 1 μg of *mneoI*/*mneoM*-tagged reporter construct. RNA was isolated 48 h post-transfection using the peqGOLD Total RNA Kit (VWR). Northern blot analysis (3 μg total RNA) was performed using the NorthernMax - Gly Kit (Thermo Scientific). *MneoI/mneoM*-tagged RNAs were detected using a biotinylated neo-sense riboprobe; L1-containing transcripts using an antisense riboprobe directed against the 5′ part of ORF2. The Thermo Scientific Biotin Chromogenic Detection Kit was used for detection. For RT-PCR 500 ng RNA were digested with DNAse I (Thermo Scientific). 250 ng of the DNAse digested RNA were subsequently reverse transcribed using the Verso cDNA synthesis kit (Thermo Scientific) and an anchored oligo dT primer containing a PCR primer binding site at its 5′ end (5′-GACCACGCGTATCGATGTCGACTTTTTTTTTTTTTTTTV-3′). Resulting cDNAs were amplified using Phusion HSII (Thermo Scientific) and a downstream primer complementary to the 5′ end of the RT primer (PCR anchor; 5′-GACCACGCGTATCGATGTCGAC-3′). H8_43_Kpn (5′- GCGGTACCTATCGAAAGCTGATGAAATGCTC-3′; H8_43_*mneoI* splice variant detection) and GS87 (5′- GCCATTGAACAAGATGGATTGCACGCAGG-3′; correct *mneoM* polyadenylation) were used as upstream primers. PCR products were cloned into pJET1.2 (Thermo Scientific) and sequenced.

### Genomic DNA isolation and characterization of H8_43 *mneoM* de novo insertions

Genomic DNA of grown-up G418-resistant colonies was isolated using the Monarch Genomic DNA Purification Kit (New England Biolabs). The 3′ ends of the insertions were determined using EPTS-LM PCR as described previously [12]. Subsequently, the de novo integrations’ 5′ ends were amplified using primers in the upstream genomic sequence.

### Generation of codon-optimized orangutan and gibbon L1 ORF1

As a basis for codon-optimization consensus sequences for the evolutionary youngest subgroups of gibbon (*N. leucogenys*) L1PA4 (L1Nomleu) and orangutan L1PA3 were generated: the sequences of all full-length L1PA3 and L1PA4 elements were retrieved using the UCSC genome browser table browser function (*P. abelii* – ponAbe3; *N. leucogenys* – nomLeu3). The sequences were aligned and, in case of orangutan, filtered manually to identify elements displaying the 129 bp 5’UTR deletion [41] characteristic for the evolutionary youngest L1PA3 subgroup. Sequences were sorted manually into subfamilies and subfamily consensus sequences were generated. Final alignments of the subfamily members to the respective subfamily consensus sequence were inspected and random mutation rates (coding sequence only; ORF1 and ORF2 assessed separately) were determined. Finally, the ORF1p consensus sequences of the subfamilies displaying the least deviation from the subfamily consensus were selected as basis for codon optimization. Codon optimization used the sequence and codon frequency of the target pBS-L1PA1-CH-mneo [26] as template. The optimized sequences were complemented with the pBS-L1PA1-CH-mneo ORF1-flanking sequences for cloning and synthesized by Thermo Scientific. The subcloned fragments obtained were transferred into pBS-L1PA1-CH-mneo yielding pBS-L1PA3/PA1-CH-mneo (orangutan) and pBS-L1PA4/PA1-CH-mneo (gibbon).

## Supplementary information

**Additional file 1: **Human and orangutan SVAs referred to in the study. Human SVA_E1: Human SVA_E elements displaying a 6 bp insertion in the SINE-R. Genomic positions, target site duplications (TSD), polymorphic status and the VNTR subunit structure are shown. Arrays of VNTR subunits are boxed. Boxes highlighted in red indicate VNTR subunits providing splice acceptors for splicing to the *mneoI* cassette. Orangutan SVAs: Orangutan SVAs genotyped and amplified. Buschi, Babu, Dunja, Kiki and Elsi are *P. abelii* individuals for which short read archives are available. Numbers (011 etc) refer to individuals genotyped on genomic DNA. Positional information refers to the primary amplicon. Fields highlighted in yellow indicate the animals from which the respective element was amplified. TSD - Target site duplication; TD – transduction. Orangutan SVA VNTR: VNTR subunit structure of the orangutan SVAs tested for their retrotranspositional activity. Arrays of VNTR subunits are boxed.

**Additional file 2: Figure S1.** Reference (hg19) and amplicon sequence of human SVA_E H8_43. Binding sites of amplification primers are highlighted in yellow; *Alu*-like domain and SINE-R are highlighted in green; the amplicon part marked in red could not be resolved using Sanger sequencing. Target site duplications are italicized and underlined. **Figure S2.** Reference and amplicon sequences of orangutan SVA OU3. Binding sites of amplification primers are highlighted in yellow; *Alu*-like domain and SINE-R in green. Target site duplications are italicized and underlined. The 3′ transduction is highlighted in grey (not included in the re-amplification product). **Figure S3.** Reference and amplicon sequences of orangutan SVA OU4. Binding sites of amplification primers are highlighted in yellow; *Alu*-like domain and SINE-R in green. Target site duplications are italicized and underlined. **Figure S4.** The minimal polyA signal used in the *mneoM* cassette facilitates correct polyadenylation of neo cDNA. 3′ RACE analysis to assess correct polyadenylation of the neomycin phosphotransferase gene using the minimal functional polyA signal [25]. The minimal polyA signal (pGL3-derived) was tested downstream of an SV40 promoter-driven neomycin phosphotransferase cDNA. The stop codon is shown in red; the polyA signal and GU-rich tract are underlined. The polyA signal mediating premature polyadenylation of elements upstream of the reporter cassette is italicized and underlined. The stop codon is shown in red; the polyA signal and GU-rich tract are underlined. The polyA signal mediating premature polyadenylation of elements upstream of the reporter cassette is italicized and underlined. **Figure S5.** Human SVA H8_43 *mneoM* de novo integrations. The L1 endonuclease cleavage site on the bottom strand is indicated in blue. Extra G residues at the 5′-ends of the insertions are shown in green; target site duplications in red. Neo – neomycin phosphotransferase gene. **Table S6.** Sequences of oligonucleotides used in amplification and re-amplification of human and orangutan SVA elements. Restriction enzyme recognition sites present in the re-amplification primers are underlined.

**Additional file 3: **Subgroups of human SVA_E elements containing both fixed and polymorphic elements. SVA_E3: VNTR subunit structure of SVA_E subgroup E3 containing four fixed and four polymorphic elements. Based on divergence from subgroup consensus (div; *Alu*-like region and SINE-R) and VNTR structure the two fixed elements on chromosome 1 would be candidates to test for activity. AF - allele frequency as provided in Stewart C, Kural D, Strömberg MP, Walker JA, Konkel MK, et al. (2011) A Comprehensive Map of Mobile Element Insertion Polymorphisms in Humans. PLoS Genet 7(8): e1002236. Arrays of VNTR subunits are boxed. Polymorphic elements are highlighted in yellow. SVA_E4: VNTR subunit structure of SVA_E subgroup E4 containing eight fixed and three polymorphic elements. Candidates to test for activity based on divergence from subgroup consensus (div; *Alu*-like region and SINE-R) and VNTR structure are indicated. AF - allele frequency; Sources: chr1:112,834,947–112,837,733–1000 Genomes Phase 3 Integrated Variant Calls track of the UCSC genome browser; chr2:223760192–223,762,683 - Stewart C, Kural D, Strömberg MP, Walker JA, Konkel MK, et al. (2011) A Comprehensive Map of Mobile Element Insertion Polymorphisms in Humans. PLoS Genet 7(8): e1002236; chr5:43,086,412-43,089,307 - dbRIP. Arrays of VNTR subunits are boxed. Polymorphic elements are highlighted in yellow.

**Additional file 4:** VNTR structure of orangutan SVA_PA__7 elements. Ten orangutan SVA_PA__7 in ponAbe3 were selected at random and their VNTR subunit structure was determined based on the code developed in Lupan et al. (2015). CONSENSUS ponAbe2 - consensus VNTR structure of the SVA_PA__7 elements identifiable in ponAbe2. The VNTR structure of the two elements tested (OU3, OU4) is given for comparison.

## Data Availability

The dataset supporting the conclusions of this article are included within the article and its additional files.

## Abbreviations

Non-LTR: Non-long terminal repeat
SVA: SINE-R-VNTR-*Alu*
LAVA: L1-*Alu*-VNTR-*Alu*
SINE-R: SINE of retroviral origin
VNTR: Variable Number of Tandem Repeats
PVA: PTGR-VNTR-*Alu*
FVA: FRAM-VNTR-*Alu*
LINE: Long Interspersed Element
myrs: Million years
ZFP: Zinc finger protein
