# Supplementary material for: LINE-1 ORF1p does not determine substrate preference for human/orangutan SVA and gibbon LAVA
Source: Mob DNA. 2020 Jul 11;11:27. doi: 10.1186/s13100-020-00222-y (PMC7353768; doi:10.1186/s13100-020-00222-y)
Supplement: Supplementary file 2 — Additional file 2: Figure S1. Reference (hg19) and amplicon sequence of human SVA_E H8_43. Binding sites of amplification primers are highlighted in yellow; Alu-like domain and SINE-R are highlighted in green; the amplicon part marked in red could not be resolved using Sanger sequencing. Target site duplications are italicized and underlined. Figure S2. Reference and amplicon sequences of orangutan SVA OU3. Binding sites of amplification primers are highlighted in yellow; Alu-like domain and SINE-R in green. Target site duplications are italicized and underlined. The 3′ transduction is highlighted in grey (not included in the re-amplification product). Figure S3. Reference and amplicon sequences of orangutan SVA OU4. Binding sites of amplification primers are highlighted in yellow; Alu-like domain and SINE-R in green. Target site duplications are italicized and underlined. Figure S4. The minimal polyA signal used in the mneoM cassette facilitates correct polyadenylation of neo cDNA. 3′ RACE analysis to assess correct polyadenylation of the neomycin phosphotransferase gene using the minimal functional polyA signal [25]. The minimal polyA signal (pGL3-derived) was tested downstream of an SV40 promoter-driven neomycin phosphotransferase cDNA. The stop codon is shown in red; the polyA signal and GU-rich tract are underlined. The polyA signal mediating premature polyadenylation of elements upstream of the reporter cassette is italicized and underlined. The stop codon is shown in red; the polyA signal and GU-rich tract are underlined. The polyA signal mediating premature polyadenylation of elements upstream of the reporter cassette is italicized and underlined. Figure S5. Human SVA H8_43 mneoM de novo integrations. The L1 endonuclease cleavage site on the bottom strand is indicated in blue. Extra G residues at the 5′-ends of the insertions are shown in green; target site duplications in red. Neo – neomycin phosphotransferase gene. Table S6. Sequences of oligonucleotides u [file 13100_2020_222_MOESM2_ESM.pdf]

Additional file 2

**Figure S1**	**Reference (hg19) and amplicon sequence of human SVA_E H8_43.**
**Figure S2**	**Reference and amplicon sequences of orangutan SVA OU3.**
**Figure S3**	**Reference and amplicon sequences of orangutan SVA OU4.**
**Figure S4**	**The minimal polyA signal used in the *mneoM* cassette facilitates correct polyadenylation of neo cDNA**
**Figure S5**	**Human SVA H8_43 *mneoM de novo* integrations.**
**Table S6**	**Sequences of oligonucleotides used in amplification and re-amplification of human and orangutan SVA elements.**

Figure S1 Reference (hg19) and amplicon sequence of human SVA_E H8_43

```
chr8:43033251-43036787  ACCTGACAAC AAACCCTAAA ATGAATCCTA CTGGAAGCAG TGGCTCCCTT TTTGGAGATC CACCAAGTCC ACATTCATAT GAAACACCAT CTAGAAGTGA CCTCCACCTC TTGCTAGTGC
Amplicon_H8_43          .......... .......... .......... .......... .......... .......... .......... .......... .......... .......... .......... ..........

chr8:43033251-43036787  CTATTAAAGT ACATATTGCC TGAGGGCTCC ATTTGGCCAT TGTCTAGATG TTTGTGTCCT CTTCTGCTAA CGACAGTTGC AAGGCCCTTG AGGACAGTGA CTAGTGCCCT CCCGACTCTG
Amplicon_H8_43          .......... .......... .......... .......... .......... .......... .......... .......... .......... .......... .......... ..........

chr8:43033251-43036787  ATACTCATTC AAAGCAGAGG GCTCACAAGC CTTCCCTCAG CTCTGCTGTC CCTGCGCACC ACTAAACTAA TGACAAAGAT ATTACTCCTT CCAGAAACCC AGCTGCCATA CAAGTGCCAG
Amplicon_H8_43          .......... .......... .......... .......... .......... .......... .......... .......... .......... .......... .......... ..........

chr8:43033251-43036787  TGAAGGTGGA TTCGTGAACT GCCGCACACT ATCGAAAGCT GATGAAATGC TCTCCCTCTC CCTCTCCCTC TCCCTCTCCC TCTCCCTCTC CCTCTCCCTC TCCCTCTCCC TCTCCCTCTC
Amplicon_H8_43          .......... .......... .......... .......... .......... .......... .......... .......... .......... .......... .......... ..........

chr8:43033251-43036787  CCTCTCCCTC TCCCTCTCCC TCTCCCTCTC CCTCTCCCTC TCCCTCTCCC TCTCCCTCTC CCTCTCCCTC TCCCTCTCCC TCCACGGTCT CCCTCTGATG CCGAGCCAAG GCTGGACGGT
Amplicon_H8_43          .......... ..-------- ---------- ---------- ---------- ---------- ---------- --........ .......... .......... .......... ..........

chr8:43033251-43036787  ACTGCTGCCA TCTCGGCTCA CTGCAACCTC CCTGCCTGAT TCTCCTGCCT CAGCCTGCCG AGTGCCTGCG ATTGCAGGCG CGCACCGCCA CGCCTGACTG GTTTTCGGTT TTTTTTGGTG
Amplicon_H8_43          .......... .......... .......... .......... .......... .......... .......... .......... .......... .......... .......T.. ..........

chr8:43033251-43036787  GAGACGGGGT TTCGCTGTGT TGGCCGGGCT GGTCTCCAGC TCCTAGCCGC GAGTGATCCG CCAGCCTCGG CCTCCCGAGG TGCCGGGATT GCAGATGGAG TCTCGTTCAC TCAGTGCTCA
Amplicon_H8_43          .......... .......... .......... .......... .......... .......... .......... .......... .......... .......... .......... ..........

chr8:43033251-43036787  ATGGTGCCCA GGCTGGAGTG CAGTGGCGTG ATCTCGGCTC GCTGCAACCA CCTCCCAGCC GCCTGCCTTG GCCTCCCGGA GAGCCGAGAT TGCAGCCTCT GCCCAGCAGC CACCCCGTCT
Amplicon_H8_43          .......... .......... .......... .......... .......... .......... .......... .......... .......... .......... .......... ..........

chr8:43033251-43036787  GGGAAGTGAG GAGCGTCTCT GCTTGGCCAC CCATCGTCTG GGATGTGAGG AGCCCCTCTG CCT GGCTGCC CAGTCTGGAA AGTGAGGAGC GTCTCTGCCC GGCCGCCATC CCATCTGGGA
Amplicon_H8_43          .......... .......... .......... .......... .......... .......... .......... .......... .......... .......... .......... ..........

chr8:43033251-43036787  AGCGAGGAGC GCCTCTTCCC CGCCGCCATC CCATCTAGGA AGTGAGGAGC GTCTCTGCCC GGCCGCCCAT CGTCTGAGAT GTGGGGAGCA CCTCTGCCCC GCCGCCCTGT CTGGGATGTG
Amplicon_H8_43          .......... .......... .......... .......... .......... .......... .......... .......... .......... .......... .......... ..........

chr8:43033251-43036787  AGGAGTGCCT CTGCTGGGCC GCAGCCCTGT CTGGGAGGTG GGGAGCGTCT CTGCCCGGCC GCTCCGTCTG AGAAGTGAGG AAACCCTCTG CCTGGCAACC GCCCCGTCTG AGAAGTGAGG
Amplicon_H8_43          .....C.... .......... .......... .......... .......... .......... .......... .......... .......... .......... .......... ..........

chr8:43033251-43036787  AGCCCCTCCG TCCGGCAACC ACCCCGTCTG GGAAATGAGG AGCGTCTCCG CCCAGCAGCC ACCCCGTCCG GGAGGGAGGT GGGGGGGGTC AGCCCCCCGC CCGGCCAGCC ACCCCGTCCG
Amplicon_H8_43          .......... ....A..... .......... .......... .......... .......... .......... .......... .......... .......... .......... G.........

chr8:43033251-43036787  GGAGGTGAGG GGCTCCTCTG CCCGGCCGCC CCTACTGGGA AGTGAGGAGC CCCTCTGCCC GGCCAGCCGC CCCATCCGGG AGGGAGGCGG TGGGGGGGGG GGTCGGCCAG CCGCCCCGTC
Amplicon_H8_43          .......... .......... .......... .......... .......... .......... .......... .......... .......... .......... .......... ..........

chr8:43033251-43036787  CGGGAGGGAG GTGGGGGGGT CAGCCCCCCG CCCGGCCAGC CGCCCCGTCT GGGAGGTGAG GGGCTCCTCT GCCCGGCCGC CCCTACTGGG AAGTGAGGAG CCCCTCTGCC CGGCCAGACG
Amplicon_H8_43          .......... .......... .......... ...C...... .......... .......... .......... .......... .......... .......... .......... ..........

chr8:43033251-43036787  CCCCGTCCAG GAGGGAGGTG GGGGGGTCAG CCCCCCGCCC GGCCAGCCGC CCAGTCCGGG AGGGAGGTGG GGGGTCAGCC CCCCGCCCGG CCAGCCGCCC CGTCTGGGAG GGAGGTGGGG
Amplicon_H8_43          .......... .......... .......... .......... .......... .......... .......... .......... .......... .......... .......... ..........

chr8:43033251-43036787  GGATCAGCCC CCCGCCTGGC CAGCCGCCCC ATCCGGGAGG TGAGGGGCGC CTCTGCCCGG CCGCCCCTAC TGGGAAGTGA GGAGCCCCTC TGCCCGGCCA GCCGCCCCGC CCGGGAGGGA
Amplicon_H8_43          .......... .......... .......... .......... .......... .......... .......... .......... .......... .......... .......... ..........

chr8:43033251-43036787  GGTGGGGGGG TCATCCCCCC ACCTGGCCAG CCGCCCCATC CGGGAGGGAG GTGGGGGG-- ---------- ---------- ---------- ---------- ---------- ----------
Amplicon_H8_43          .......... .......... .......... .......... .......... ........GT CAGCCCCCCG CCCGGCCAGC CGCCCCGTCC GGGAGGGAGG TGGGGGGGGT CAGCCCCCCG

chr8:43033251-43036787  ---------- ---------- ---------- -------ATC AGCCCCCCGC CTGGCCAGTC GCCCCGTCCG GGAGGTGAGG GGCGCCTCTG CCCGGCCGCC CCTACTGGAA AGTGAGGAGC
Amplicon_H8_43          CCCGGCCAGC CGCCCCGTCC GGGAGGGAGG TGGGGGG... .......... .......... .......... .......... .......... .......... .......... ..........

chr8:43033251-43036787  CCCTCTGTCC GGCCAGCCGC CCCGTCCGGG AGGGAGGCGG TGGGGGGGGT CGGCCAGCCG CCCCGTCCGG GAGGGAGGTG GGGGGGGTCA GCCCCCCTTC CGGCCGGCCG CCCCGTCCGG
Amplicon_H8_43          .......... .......... .......... .......... .......... .......... .......... .......... .......... .......... .......... ..........

chr8:43033251-43036787  GAGGTGAGGG GCGCCTCTGC CCGGCCGCCC CTACTGGGAA GTGAGGACCC CTCTGCCCGG CCAGCCGCCC CGTCCGGGAG GGAGGTGGGG GGGACAGCCC CCCGCCCAGC CAGCCGCCCT
Amplicon_H8_43          .......... .......... .......... .......... .......... .......... .......... .......... .......... .......... .......... ..........

chr8:43033251-43036787  ATCCAGGAGG TGAGGGGCGC CTCTGCCCGG CCGCCCCTAC TGGGAAGTGA GGAGCCCCTC TGCCTGGCCA GCCGCCCCGT CCGGGAGGGT GGTGGGGGGG TCAGCCCCCG CCCGGCCAGC
Amplicon_H8_43          .......... .......... .......... .......... .......... .......... .......... .......... .......... .......... .......... ..........
```

chr8:43033251-43036787 CGCCCCATCC GGGAGGTGAG GGGCGCTTCT GCCCGGCCGC CCCTACTGGG AAGTGAGGAG CCCCTCTGCC CGGCCACGAC CCCGTCTGGG AGGTGTGCCC AGCGGCTCAT TGGGGATGGG
Amplicon_H8_43 ....

chr8:43033251-43036787 CCATGATGAC AATGGCGGTT TTGTGGAATA GAAAGGCGGG AAGGGTGGGG AAAAAATTGA GAAATCGGAT GGTTGCCGGG TCTGTGTGGA TAGAAGTAGA CATGGGAGAC TTTTCATTTT
Amplicon_H8_43 ....

chr8:43033251-43036787 GTTCTGTACT AAGAAAAATT CTTCTGCCTT GGGATCCTGT TGATCTGTGA CCTTATCCCC AACCCTGTGC TCTCTGAAAC ATGTGCTGTG TCCACTCAGG GTTAAATGGA TTAAGGGTGG
Amplicon_H8_43 ....

chr8:43033251-43036787 TGCAAGATGT GCTTTGTTAA ACAGATGCTT GAAGGCAGCA TGCTCGTTAA GAGTCATCAC CACTCCCTAA TCTTAAGTAC CCAGGGACAC AAACACTGCG GAAGGCCAAG GCCGCAGGGT
Amplicon_H8_43 ....

chr8:43033251-43036787 CCTCTGCCTA GGAAAACCAG AGACCTTTGT TCACTTGTTT ATCTGCTGAC CTTCCCTCCA CTATTGTCCT ATGACCCTGC CAAATCCCAA CACCCAAGAA TGATCAATAA AAAATAAATA
Amplicon_H8_43 ....

chr8:43033251-43036787 AATAAATTTA AAAAAAAAAA AAAAAAAAAG ***AAAGCTGATG AAAT***CACATT AGCAGGGGGC AAAGTTGGAG ACCAAGGCGG CCTGGTCTGG TCCCTGCTGT GCCTCTCACT ACACTCAGAT
Amplicon_H8_43 ....

chr8:43033251-43036787 AATCCAGAGA AAACCTTCAA TCGCTCTTGG CCTCAGACTT TTCCTCTGCA CAAGAGAAGG GTTTGAGTAG TCTCAGAGGC TCCTCCCAGG TCTAAAATTC AAAAATAATA ATAATAATGC
Amplicon_H8_43 ....

chr8:43033251-43036787 TCACAGTTAG ACAACCATTG TATATACTTT CCTAATGATT ATTCTATTTG CAGGCACCTG AGATGGAGGA ATTGCATTTC TCTCAAATTA CAACATATAG TTTCCATATT TCAACCTGAA
Amplicon_H8_43 ....

chr8:43033251-43036787 TATATATAAC AGAGGGAAAA AGTACTTACA TGGACCAAGG CAATAATTGG GATTCACAAT GATAATTCCT ATGCAGATTA ACAGGAAACT CCTCCATGCA ATCTTCCCCA GCAATCTGAA
Amplicon_H8_43 ....

chr8:43033251-43036787 TTTTGAACAC CCCCGTTGCA GTATAGAAGT CATCGA
Amplicon_H8_43 ....

Binding sites of amplification primers are highlighted in yellow; *Alu*-like domain and SINE-R are highlighted in green; the amplicon part marked in red could not be resolved using Sanger sequencing. Target site duplications are italicized and underlined.

Figure S2 **Reference and amplicon sequences of orangutan SVA OU3.**

```
ponAbe2       GCAAATACGG TTGTAAACAC AGGTGAAATT TTAAATCACT TCTGAGGAAG AAAATAACAT AATGGAATTC CACACCTTTT TTGAGGATAA GATGTGCATA TGCTTCAACT ATGAAAACGG TAGACAATGT
ponAbe3       .......... .......... .......... .......... .......... .......... .......... .......... .......... .......... .......... .......... ..........
Amplicon_OU3  .......... .......... .......... .......... .......... .......... .......... .......... .......... .......... .......... .......... ..........

ponAbe2       TGTCCAGAAA TGGCATGTAT CAGCTGCTCT TGAGAAATAA AAATAAAATT CTTCTCCCTC TCCCTCTCCC TCTCC----- ---------- ---------- ---------- ---------- ---CTCTCCC
ponAbe3       .......... .......... .......... .......... .......... .......... .......... .....CTCTC CCTCTCCCTC TCCCTCTCCC TCTCCCTCTC CCTCTCCCTC TCC.......
Amplicon_OU3  .......... .......... .......... .......... .......... .......... .......... .....----- ---------- ---------- ---------- ---------- ---.......

ponAbe2       TCTCCCTCTC CCTCTCTTTT TCGGTCTCCC TCTCCTTCTT TTTTCGGTCT CCCTCTGTTG CCGAAGCTGG ACTGTACTGC CGGGATCTCG GCTTGCTGCA ACCTCCCTGC CTCGGGCTCC TGTGACTCTC
ponAbe3       .......... .......... .......... .......... .......... .......... .......... .......... .......... .......... .......... .......... ..........
Amplicon_OU3  .......... .......... .......... .......... .......... .......... .......... .......... .......... .......... .......... .......... ..........

ponAbe2       CTGCCTCGGC CTGCCGAGTG CCTGGGATTG CAGGCGCGCG CTGCCACGCC TGAATGGTTT TTGTATTTTT GGTGGAGATG GGGTTTCGCC GTGTTGACCA GGCTGGTCTC CAGCTCCTGG CCTCGAGTGA
ponAbe3       .......... .......... .......... .......... .......... .......... .......... .......... .......... .......... .......... .......... ..........
Amplicon_OU3  .......... .......... .......... .......... .......... .......... .......... .......... .......... .......... .......... .......... ..........

ponAbe2       TCTGCCTGCC TCGGCCTCCC GAGGTGCTGG GATTGCAGAC GGAGTCTCGC TAACTCAATG CTCAATGGTG CTCAGGCTGG AGTGCAGTGG TGTGATCTCG GCTCTCTGCA ACCTCCACCT ACCAGCCTCC
ponAbe3       .......... .......... .......... .......... .......... .......... .......... .......... .......... .......... .......... .......... ..........
Amplicon_OU3  .......... .......... .......... .......... .......... .......... .......... .......... .......... .......... .......... .......... ..........

ponAbe2       TGCCTTGGCC TCTTAAAGTG CTAAGATTAC AGCCTCTGCC CCACCGCCAC CCCGTCTAGG AAGTGAGGAG CGTCTCTGCC TGGCCGCCCA TCGTCTGGGA TGTGAGGAGC CCCTCTGCCC GGCCGCCCTA
ponAbe3       .......... .......... .......... .......... .......... .......... .......... .......... .......... .......... .......... .......... ..........
Amplicon_OU3  .......... .......... .......... .......... .......... .......... .......... .......... .......... .......... .......... .......... ..........

ponAbe2       TCTGGGAAGT GAGGAGCGCC TCTGCCCGGC CGCCCATCAT CTGGGATGTG AGGAGCGCCT CTGCCCGGCT GCCACCCTGT CTGGGAGGAA GTGAGGAGCG CCTCTGCCCG GCTGCCCCGT CTGGGAGATG
ponAbe3       .......... .......... .......... .......... .......... .......... .......... .......... .......... .......... .......... .......... ..........
Amplicon_OU3  .......... .......... .......... .......... .......... .......... .......... .......... .......... .......... .......... .......... ..........

ponAbe2       AGGAGCACCT CTGCCCGGCC GCCCCGTCTG GGAGGAAGTG AGGAGCGCCT CTGCCCTGTT GCCCTATCTG GGAAGTGAGG AGCGCCTCTG CCTGGCCGCC ACCCCGTCTG GGAAGTGAGG AGCGCCTCTG
ponAbe3       .......... .......... .......... .......... .......... .......... .......... .......... .......... .......... .......... .......... ..........
Amplicon_OU3  .......... .......... .......... .......... .......... .......... .......... .......... .......... .......... .......... .......... ..........

ponAbe2       CCCGGCTGCC ACCCCATATG GGAAGTGAGG AGCGCCTCTG CCCAGCCGCC CCTTCTGGGA GGTGAAGAGC GCCTCTGCCC AGCCGCCCCT TCTGGGAGGT GAGGAGTGCC TCTGCCCGGC CGCCCCGTCT
ponAbe3       .......... .......... .......... .......... .......... .......... .......... .......... .......... .......... .......... .......... ..........
Amplicon_OU3  .......... .......... .......... .......... .......... .......... .......... .......... .......... .......... .......... .......... ..........

ponAbe2       GGGAGGTGAG GAGCGCCTCT GCCTGGCCGC CACCCCGTCT GGGAGGAAGT GAGGAGCACC TCTGCCCAGC TGCCCCATCT GGGAAGTGAG GAGCGCCTCT GCCCGGCTGC CACTCCCTAT GGGAAGTGAG
ponAbe3       .......... .......... .......... .......... .......... .......... .......... .......... .......... .......... .......... .......... ..........
Amplicon_OU3  .......... .......... .......... .......... .......... .......... .......... .......... .......... .......... .......... .......... ..........

ponAbe2       GAGCGCCTCT GCCCAGCCGC CCACTCTGGG AAGTGCGGAG CGCCTCTGCC CGGCCGCCCA CTCTGGGAAG TGAGGAGCGC CTCTGCCCGG CCGCCCACTC TGGGAGGTGA GGAGTGCCTC TGCCCGGCCG
ponAbe3       .......... .......... .......... .......... .......... .......... .......... .......... .......... .......... .......... .......... ..........
Amplicon_OU3  .......... .......... .......... .......... .......... .......... .......... .......... .......... .......... .......... .......... ..........

ponAbe2       CCCCGTCTGG GAGGTGAGGA GCGCCTCTGC CTGGCCGCCA CCCCGTCTGG GAGGAAGTGA GGAGCACCTC TGCCCAGCCG CCCACTCTGG GAGGTGAGGA GCGCCTCTGC CTGGCCACTC CGTCTGGGAA
ponAbe3       .......... .......... .......... .......... .......... .......... .......... .......... .......... .......... .......... .......... ..........
Amplicon_OU3  .......... .......... .......... .......... .......... .......... .......... .......... .......... .......... .......... .......... ..........

ponAbe2       GGGAGGAGCG CCTCTGCCCG GCCACCCCAT CTGGGAGGTG AGGAGCGCCT CTGCCCGGCT GCCACCCCGT CTGGGAGGAA GTGAGGAGCA CCTCTGCCCA GCTGCCCCAT CTGGGAAGTG AGGAGCGCCT
ponAbe3       .......... .......... .......... .......... .......... .......... .......... .......... .......... .......... .......... .......... ..........
Amplicon_OU3  .......... .......... .......... .......... .......... .......... .......... .......... .......... .......... .......... .......... ..........

ponAbe2       CTGCCCGGCC GCCACCCCAT ATGGGAAGTG AGGAGCGCCT CTGCCTGACC ACTCCGTCTG GGAGGTGAGG AGCGCCTCTG CCCAGCCGCC CCGTCTGGGA GGTGAGGAGT GCCTCTGCCC GGCCGTCACC
ponAbe3       .......... .......... .......... .......... .......... .......... .......... .......... .......... .......... .......... .......... ..........
Amplicon_OU3  .......... .......... .......... .......... .......... .......... .......... .......... .......... .......... .......... .......... ..........

ponAbe2       CCGTCTGGGA GGAAGTGAGG AGCACCTCTG CCCGGCTGCC CCGTCTGGGA GATAAGGAGC ACCTCTGCCC GGCCGCCCCG TCTGGGAGAT GAGGAGCACC TCTGCCCGGC CGCCCCGTCT GGGAGGTGGG
ponAbe3       .......... .......... .......... .......... .......... .......... .......... .......... .......... .......... .......... .......... ..........
Amplicon_OU3  .......... .......... .......... .......... .......... .......... .......... .......... .......... .......... .......... .......... ..........

ponAbe2       GAGTGCCTCT GCCCGGCTGC CACCCCGTCT GGGAGGAAGT GAGGAGCACC TCTGCCCGGC CGCCCCCTCT GGGAAGTGAG GAGCGCCTCT GCCTGGCCGC CACCCCGTCT GGGAGGAAGT GAGGAGCGAC
ponAbe3       .......... .......... .......... .......... .......... .......... .......... .......... .......... .......... .......... .......... ..........
Amplicon_OU3  .......... .......... .......... .......... .......... .......... .......... .......... .......... .......... .......... .......... ..........
```

```
ponAbe2       TCTGCCTGGC TGCCCCATCT GGGAAGGGAG GAACACCTCT GCCCGGCCGC CACACCGTCT GGGAAGTGAG GAGCGCCTCT GCCTGGCTGC CCCATCTGGG AAGGGAGGAG CACCTCTGCC CAGCCGCCAC
ponAbe3       .......... .......... .......... .......... .......... .......... .......... .......... .......... .......... .......... .......... ..........
Amplicon_OU3  .......... .......... .......... .......... .......... .......... .......... .......... .......... .......... .......... .......... ..........

ponAbe2       ACCGTCTGGG AAGTGAGGAG CGCCTCTGCC TGGTCGCCCC GTCTAGGAGG TGAGGAACGC CTCTGCCCGG CCGCCCAGTC TGGGAAGTGA GGAGCGCCTC TGCCCGGCCG CCCTGTCTGG GAGGTGAGGA
ponAbe3       .......... .......... .......... .......... .......... .......... .......... .......... .......... .......... .......... .......... ..........
Amplicon_OU3  .......... .......... .......... .......... .......... .......... .......... .......... .......... .......... .......... .......... ..........

ponAbe2       GCGCCTCTGC CTGGCCGCCA CCCCATCTGG GAGGAAGTGA GGAGCGTCTC TGCCCGGCCG CCCCGTCTGG GAAGTGAGGA GCGCCTCTGC CCGGCCGCCC CCTCTGGGAA GTGAGGAGCG CCTCTGCTCG
ponAbe3       .......... .......... .......... .......... .......... .......... .......... .......... .......... .......... .......... .......... ..........
Amplicon_OU3  .......... .......... .......... .......... .......... .......... .......... .......... .......... .......... .......... .......... ..........

ponAbe2       GCCGCCCCCT CTGGGAAGTG AGGAGCGCCT CTGCTCGGCC GCCCCGTCGG GGAAGTGAGG AGCGCCTCTG CCCGGCCGCC CCGTCTGGGA GGTGAGGAGC GCCTCTGCCC GGCTGCCACC CGGTCTGGGA
ponAbe3       .......... .......... .......... .......... .......... .......... .......... .......... .......... .......... .......... .......... ..........
Amplicon_OU3  .......... .......... .......... .......... .......... .......... .......... .......... .......... .......... .......... .......... ..........

ponAbe2       GGAACTGAGG AGCGCCTCTG CCCGGGCGGC CCCGTCTGGG AAGCGAGGAG CGCCTCTGCC CGGGCGGCCC CGTCGGGGAA GTGAGGAGCG CCTCTGCCCG GCCGCCCCGT CTGGGAGGAG AGGAGCGCCT
ponAbe3       .......... .......... .......... .......... .......... .......... .......... .......... .......... .......... .......... .......... ..........
Amplicon_OU3  .......... .......... .......... .......... .......... .......... .......... .......... .......... .......... .......... .......... ..........

ponAbe2       CTGCCCGGGC GGCCCCGTCT GGGAAGCGAG GGGCGCCTCT GCCCAGCCGC CCTGTCTGGG AGGTGAGGAG CGCCTCTGCC CGGCTGCCCT GTCTGGGAGG TGTACCCAAC AGCTCCGAAG AGACAGCGAC
ponAbe3       .......... .......... .......... .......... .......... .......... .......... .......... .......... .......... .......... .......... ..........
Amplicon_OU3  .......... .......... .......... .......... .......... .......... .......... .......... .......... .......... .......... .......... ..........

ponAbe2       CATCGGGAGC GGGCCATGAG GACGATGGCG GTTTTGTTGA AGAGAAGGGG AGGAAGTGTG GGGAAAGGAA GGAGAGATCA GATTGTTGCT GTGTCTGTGT AGAAAGGGGT GGGCATAGGA GACTCCATTT
ponAbe3       ......-... .......... .......... .......... .......... .......... .......... .......... .......... .......... .......... .......... ..........
Amplicon_OU3  .......... .......... .......... .......... .......... .......... .......... .......... .......... .......... .......... .......... ..........

ponAbe2       TGTTCTGACT AGGAGAAATT CTTCTGCCTT GGGATGCTGT TGATCTATGG CCTTTCCCCC AGCCCCCTGC TCTCTGAAAC ATGTGCTGTG TCAACTCAGG GTTAAATGGA TTAAGGGTGG TGCAAGATGT
ponAbe3       .......... .......... .......... .......... .......... .......... .......... .......... .......... .......... .......... .......... ..........
Amplicon_OU3  .......... .......... .......... .......... .......... .......... .......... .......... .......... .......... .......... .......... ..........

ponAbe2       GCTTTGTTAA ACAGATGCTT GAAGGCAGCA TGCTCTTTAA GAGTCATCAC CACTCCCTAA TCTCAAGTAC TCAGGGGCAC AAACACTGCA GAAGGCCGCA GGGTCCTCTG CCTAGGAAAA CCAGAGACCT
ponAbe3       .......... .......... .......... .......... .......... .......... .......... .......... .......... .......... .......... .......... ..........
Amplicon_OU3  .......... .......... .......... .......... .......... .......... .......... .......... .......... .......... .......... .......... ..........

ponAbe2       TTGTTCATGT GTTTATCTCC TGACCTTCTC TCCACTATTA TCCTATGACC CTGCCATATC CCCCTCTCCG AGAAACACCC AAGAATGATC AATAAATACT TCAGAAATTA AAAAAAAAAA AAAAAAAAAG
ponAbe3       .......... .......... .......... .......... .......... .......... .......... .......... .......... .......... .......... .......... ..........
Amplicon_OU3  .......... .......... .......... .......... .......... .......... .......... .......... .......... .......... .......... .......... ..........

ponAbe2       AAATCCATGA TTCTACCATT GAAGGAAACC ACATTTTGAT ACATTTCTTT CTAGTCATTT TCTGAGCATA TTTCTGTTTA CATTATTAAA ATCATGCTGT ATGTAATGCT TGAATTGATT TTTTTCAGCT
ponAbe3       .......... .......... .......... .......... .......... .......... .......... .......... .......... .......... .......... .......... ..........
Amplicon_OU3  .......... .......... .......... .......... .......... .......... .......... .......... .......... .......... .......... .......... ..........

ponAbe2       TTATTACGCT ATGATCATCA AAAATCATAT AAAGTGAACA ACTCGAAAAA AAAAAAAAAT AAAAATAAAA TAAAATAAAA TAAAATTCTA AGCACCCCCT AACTGACTGA ATGGATGC
ponAbe3       .......... .......... .......... .......... .......... .......... .......... .......... .......... .......... .......... ........
Amplicon_OU3  .......... .......... .......... .......... .......... .......... .......... .......... .......... .......... .......... ........
```

Binding sites of amplification primers are highlighted in yellow; *Alu*-like domain and SINE-R in green. Target site duplications are italicized and underlined. The 3’ transduction is highlighted in grey (not included in the re-amplification product).

Figure S3 Reference and amplicon sequences of orangutan SVA OU4

```
ponAbe2       AACATAGGTA ACATGGCACT GGCCTTCTCT CAATAAAGAA TTCTGATTCT TCATAAGAAC CTGGGCTGAT GAAAAAGCCA AACGGCTCGG TAGAATTGTG ATATTTATTT CCCAAGTGCT TCCATTTCTA
ponAbe3       .......... .......... .......... .......... .......... .......... .......... .......... .......... .......... .......... .......... ..........
Amplicon_OU4  .......... .......... .......... .......... .......... .......... .......... .......... .......... .......... .......... .......... ..........

ponAbe2       GCCAAGCCTC TTAGAATACA AGCTTTCTGC CTCTGCCTCT CCCCCTCCCC CTCCCCCTCC CCCTCCCTCT CCCCTCTTTT TTCGGTCTCC CTCTCCTTCT TTTTTCGGTC TCCCTCTGTT GCCGAAGCTG
ponAbe3       .......... .......... .......... .......... .......... .......... .......... .......... .......... .......... .......... .......... ..........
Amplicon_OU4  .......... .......... .......... .......... .......... .......... .......... .......... .......... .......... .......... .......... ..T.......

ponAbe2       GACTGTACTG CCGGGATCTC GGCTTGCTGC AACCTCCCTG CCTCGGGCTC CTGTGACTCT CCTGCCTTGG CCTGCCGAGT GCCTGGGATT GCAGGCGCGC GCCGCCACGC CTGAATGGTT TTTGTATTTT
ponAbe3       .......... .......... .......... .......... .......... .......... .......... .......... .......... .......... .......... .......... ..........
Amplicon_OU4  .......... .......... .......... .......... .......... .......... .......... .......... .......... .......... .......... .......... ..........

ponAbe2       TGGTGGAGAC GGGGTTTCGC CGTGTTGACC GGGCTGGTCT CCAGCTCCTG GCCTCGAGTG ATCTGCCTGC CGCGGCCTCC CGAGGTGCTG GGATTGCAGA CGGAGTCTCG CTAACTCAAT GCTCAATGGT
ponAbe3       .......... .......... .......... .......... .......... .......... .......... .......... .......... .......... .......... .......... ..........
Amplicon_OU4  .......... .......... .......... .......... .......... .......... .......... .......... .......... .......... .......... .......... ..........

ponAbe2       GCTCAGGCTG GAGTGCAGTG GTGTGATCTC GGCTCTCTGC AACCTCCACC TACCAGCCTC CTGCCTTGGC CTCTTAAAGT GCTAAGATTA CAGCCTCTGC CCCACCGCCA CCCCGTCTAG GAAGTGAGGA
ponAbe3       .......... .......... .......... .......... .......... .......... .......... .......... .......... .......... .......... .......... ..........
Amplicon_OU4  .......... .......... .......... .......... .......... .......... .......... .......... .......... .......... .......... .......... ..........

ponAbe2       GCGTCTCTGC CTGGCCGCCC ATCGTCTGGG ATGTGAGGAG CCCCTCTGCC CGGCCGCCCT ATCTGGGAAG TGAGGAGCGC CTCTGCTCGG CCGCCCATCA TCTGGGATGT GAGGAGCGCC TCTGCCCGGC
ponAbe3       .......... .......... .......... .......... .......... .......... .......... .......... .......... .......... .......... .......... ..........
Amplicon_OU4  .......... .......... .......... .......... .......... .......... .......... .......... .......... .......... .......... .......... ..........

ponAbe2       TGCCACCCCG TCTGGGAGGA AGTGAGGAGC GCCTCTGCCC GGCTGCCCCG TCTGGGAGAT GAGGAGCACC TCTGCCCGGC CGCCCCGTCT GGGAGGAAGT GAGGAGCGCC TCTGCCCTGT TGCCCTATCT
ponAbe3       .......... .......... .......... .......... .......... .......... .......... .......... .......... .......... .......... .......... ..........
Amplicon_OU4  .......... .......... .......... .......... .......... .......... .......... .......... .......... .......... .......... .......... ..........

ponAbe2       GGGAAGTGAG GAGCGCCTCT GCCTGGCCGC CACCCCGTCT GGGAAGTGAG GAGCGCCTCT GCCCGGCTGC CACCCCATAT GGGAAGTGAG GAGCGCCTCT GCCCAGCCGC CCCTTCTGGG AGGTGAGGAG
ponAbe3       .......... .......... .......... .......... .......... .......... .......... .......... .......... .......... .......... .......... ..........
Amplicon_OU4  .......... .......... .......... .......... .......... .......... .......... .......... .......... .......... .......... .......... ..........

ponAbe2       CGCCTCTGCC CAGCCGCCCC GTCTGGGAGG CGAGGAGTGC CTCTGCCCGG CCGCCCCGTC TGGGAGGTGA GGAGCGCCTC TGCCTGGCCG CCACCCCGTC TGGGAGGAAG TGAGGAGCCC CTCTGCCCAG
ponAbe3       .......... .......... .......... .......... .......... .......... .......... .......... .......... .......... .......... .......... ..........
Amplicon_OU4  .......... .......... .......... .......... .......... .......... .......... .......... .......... .......... .......... .......... ..........

ponAbe2       CTGCCCCATC TGGGAAGTGA GGAGCGCCTC TGCCCGGCTG CCCCCCCTT- ---------- ---------- ---------- ---------- ---------- ---------- ---------- ----------
ponAbe3       .......... .......... .......... .......... ..A....C.A TGGGAAGTGA GGAGCGCCTC TGCCCGGCCG CCCACTCTGG GAAGTGAGGA GCGCCTCTGC CCGGCCGCCC ACTCTGGGAA
Amplicon_OU4  .......... .......... .......... .......... ..A....C.A TGGGAAGTGA GGAGCGCCTC TGCCCGGCCG CCCACTCTGG GAAGTGAGGA GCGCCTCTGC CCGGCCGCCC ACTCTGGGAA

ponAbe2       ---------- ---------- ---------- ---------- ---------- ---------- ---------- ---------- ---------- ---------- ---------- ---------- ----------
ponAbe3       GTGAGGAGCG CCTCTGCCCG GCCGCCCACT CTGGGAGGTG AGGAGTGCCT CTGCCCG-CC GCCCCGTCTG GGAGGTGAGG AGCGCCTCTG CCTGGCCGCC ACCCCGTCTG GGAGGAAGTG AGGAGCGCCT
Amplicon_OU4  GTGAGGAGCG CCTCTGCCCG GCCGCCCACT CTGGGAGGTG AGGAGTGCCT CTGCCCGGCC GCCCCGTCTG GGAGGTGAGG AGCGCCTCTG CCTGGCCGCC ACCCCGTCTG GGAGGAAGTG AGGAGCGCCT

ponAbe2       ---------- ---------- ---------- ---------- ---------- ---------G GGAAGTGAGG AGCGCCTCTG CCCGGCTGCC ACCCCCTATG GGAAGTGAGG AGCGCCTCTG CCCGGCCGCC
ponAbe3       CTGCCTGGCC GCCCCGTCTG GGAGGAAGTG AGGAGCACCT CTGCCCAGCT GCCCCATCT. .......... .......... .......... .....-.... .......... .......... ..........
Amplicon_OU4  CTGCCTGGCC GCCCCGTCTG GGAGGAAGTG AGGAGCACCT CTGCCCAGCT GCCCCATCT. .......... .......... .......... .......... .......... .......... ..........

ponAbe2       CACTCTGGGA AGTGAGGAGC GCCTCTGCCC GGCCGCCCAC TCTGGGAAGT GAGGAGCGCC TCTGCCCGGC CGCCCACTCT GGGAGGTGAG GAGTGCCTCT GCCCGGCCGC CCCGTCTGGG AGGTGAGGAG
ponAbe3       .......... .......... .......... .......... .......... .......... .......... .......... .......... .......... .......... .......... ..........
Amplicon_OU4  .......... .......... .......... .......... .......... .......... .......... .......... .......... .......... .......... .......... ..........

ponAbe2       CGCCTCTGCC TGGCCGCCAC CCCGTCTGGG AGGAAGTGAG GAGCACCTCT GCCCGGCCGC CCACTCTGGG AGGTGAGGAG CGCCTCTGCC TGGCCACTCC GTCTGGGAAG GGAGGAGCGC CTCTGCCCGG
ponAbe3       .......... .......... .......... .......... .......... .......... .......... .......... .......... .......... .......... .......... ..........
Amplicon_OU4  .......... .......... .......... .......... .......... .......... .......... .......... .......... .......... .......... .......... ..........

ponAbe2       CCACCCCGTC TGGGAGGTGA GGAGCGCCTC TGCCCGGCTG CCACCCCGTC TGGGAGGAAG TGAGGAGCAC CTCTGCCCGG CTGCCCCATC TGGGAAGTGA GGAGCGCCTC TGCCCGGCCG CCACCCCATA
ponAbe3       ......-... .......... .......... .......... ......-... .......... .......... .......... .......... .......... .......... .......... ..........
Amplicon_OU4  .......... .......... .......... .......... .......... .......... .......... .......... .......... .......... .......... .......... ..........

ponAbe2       TGGGAAGTGA GGAGCGCCTC TGCCTGACCA CTCCGTCTGG GAGGTGAGGA GCGCCTCTGC CCGGCCGCCC CGTCTGGGAG GTGAGGAGTG CCTCTGCCCG GCCGTCACCC CGTCTGGGAG GAAGTGAGGA
ponAbe3       .......... .......... .......... .......... .......... .......... .......... .......... .......... .......... .......... .......... ..........
Amplicon_OU4  .......... .......... .......... .......... .......... .......... .......... .......... .......... .......... .......... .......... ..........
```

```
ponAbe2       GCACCTCTGC CCGGCTGCCC CGTCTGGGAG ATGAGGAGCA CCTCTGCCCG GCCGCCCCGT CTGGGAGATG AGGAGCACCT CTGCCCGGCC GCCCCGTCTG GGAGGTGAGG AGTGCCTCTG CCCGGCTGCC
ponAbe3       .......... .......... .......... .......... .......... .......... .......... .......... .......... .......... .......... .......... ..........
Amplicon_OU4  .......... .......... .......... .......... .......... .......... .......... .......... .......... .......... .......... .......... ..........

ponAbe2       ACCCCGTCTG GGAGGAAGTG AGGAGCGCCT CTGCCTGGCT GCCCCATCTG GGAAGGGAGG AGCACCTCTG CCCAGCCGCC ACACCGTCTG GGAAGTGAGG AGCGCCTCTG CCTGGTCGCC CCGTCTAGGA
ponAbe3       .......... .......... .......... .......... .......... .......... .......... .......... .......... .......... .......... .......... ..........
Amplicon_OU4  .......... .......... .......... .......... .......... .......... .......... .......... .......... .......... .......... .......... ..........

ponAbe2       GGTGAGGAGC GCCTCTGCCC GGCCGCCCAG TCTGGGAAGT GAGGAGTGCC TCTGCCCGGC CGCCCTGTCT GGGAGGTGAG GAGCGCCTCT GCCTGGCCGC CACCCCATCT GGGAGGAAGT GAGGAGCGTC
ponAbe3       .......... .......... .......... .......... .......... .......... .......... .......... .......... .......... .......... .......... ..........
Amplicon_OU4  .......... .......... .......... .......... .......... .......... .......... .......... .......... .......... .......... .......... ..........

ponAbe2       TCTGCCCAGC CGCCCCGTCT GGGAAGTGAG GAGCGCCTCT GCCCGGCCGC CCCCTCTGGG AAGTGAGGAG CGCCTCTGCT CGGCCGCCCC CTCTGGGAAG TGAGGAGCGC CTCTGCTCGG CCGCCCCGTC
ponAbe3       .......... .......... .......... .......... .......... .......... .......... .......... .......... .......... .......... .......... ..........
Amplicon_OU4  .......G.. .......... .......... .......... .......... .......... .......... .......... .......... .......... .......... .......... ..........

ponAbe2       GGGGAAGTGA GGAGCGCCTC TGCCCGGCCG CCCCGTCTGG GAGGTGAGGA GCGCCTCTGC CCGGCTGCCA CCCGGTCTGG GAGGAACTGA GGAGCGCCTC TGCCCGGGCG GCCCCGTCTG GGAAGCGAGG
ponAbe3       .......... .......... .......... .......... .......... .......... .......... .......... .......... .......... .......... .......... ..........
Amplicon_OU4  .......... .......... .......... .......... .......... .......... .......... .......... .......... .......... .......... .......... ..........

ponAbe2       AGCGCCTCTG CCCGGGCGGC CCCGTCGGGG AAGTGAGGAG CGCCTCTGCC CGGCCGCCCC GTCTGGGAGG AGAGGAGCGC CTCTGCCCGG GCGGCCCCGT CTGGGAAGCG AGGGGCGCCT CTGCCCAGCC
ponAbe3       .......... .......... .......... .......... .......... .......... .......... .......... .......... .......... .......... .......... ..........
Amplicon_OU4  .......... .......... .......... .......... .......... .......... .......... .......... .......... .......... .......... .......... ..........

ponAbe2       GCCCTGTCTG GGAGGTGAGG AGCGCCTCTG CCCGGCTGCC CTGTCTGGGA GGTGTACCCA ACAGCTCCGA AGAGACAGCG ACCATCGGGA GCGGGCCATG AGGACGATGG CGGTTTTGTT GAAGAGAAGG
ponAbe3       .......... .......... .......... .......... .......... .......... .......... .......... .......... .......... .......... .......... ..........
Amplicon_OU4  .......... .......... .......... .......... .......... .......... .......... .......... .......... .......... .......... .......... ..........

ponAbe2       GGAGGAAGTG TGGGGAAAGG AAGGAGAGAT CAGATTGTTG CTGTGTCTGT GTAGAAAGGG GTGGGCATAG GAGACTCCAT TTTGTTCTGA CTAGGAGAAA TTCTTCTGCC TTGGGATGCT GTTGATCTAT
ponAbe3       .......... .......... .......... .......... .......... .......... .......... .......... .......... .......... .......... .......... ..........
Amplicon_OU4  .......... .......... .......... .......... .......... .......... .......... .......... .......... .......... .......... .......... ..........

ponAbe2       GGCCTTTCCC CCAGCCCCCT GCTCTCTGAA ACATGTGCTG TGTCAACTCA GGGTTAAATG GATTAAGGGT GGTGCAAGAT GTGCTTTGTT AAACAGATGC TTGAAGGCAG CATGCTCTTT AAGAGTCATC
ponAbe3       .......... .......... .......... .......... .......... .......... .......... .......... .......... .......... .......... .......... ..........
Amplicon_OU4  .......... .......... .......... .......... .......... .......... .......... .......... .......... .......... .......... .......... ..........

ponAbe2       ACCACTCCCT AATCTCAAGT ACTCAGGGGC ACAAACACTG CAGAAGGCCG CAGGGTCCTC TGCCTAGGAA AACCAGAGAC CTTTGTTCAT GTGTTTATCT CCTGACCTTC TCTCCACTAT TATCCTATGA
ponAbe3       .......... .......... .......... .......... .......... .......... .......... .......... .......... .......... .......... .......... ..........
Amplicon_OU4  .......... .......... .......... .......... .......... .......... .......... .......... .......... .......... .......... .......... ..........

ponAbe2       CCCTGCCATA TCCCCCTCTC CGAGAAACAC CCAAGAATGA TCAATAAATA CTTCAGAAAT TTAAAAAAAA AAAAAGAAAA CCTCAGTAAG TCCTAAAAAG TAAAAAAAAA AAAAAA-AGA ATACAAGCTT
ponAbe3       .......... .......... .......... .......... .......... .......... .......... .......... .......... .......... .......... .....--A.. ..........
Amplicon_OU4  .......... .......... .......... .......... .......... .......... .......... .......... .......... .......... .......... .......A.. ..........

ponAbe2       TCTTTATATT CAATTTATAA GAGTCAAGCT ATGTTGCCCA GGCTGGTCTT GAACTCCTGG CCTCAAGTGA TCCTCCTGCC TCAGCCTCCC AAAGTGCTGG GATTCCAGGT GAAAGCCACC AAGCCCAGTC
ponAbe3       .......... .......... .......... .......... .......... .......... .......... .......... .......... .......... .......... .......... ..........
Amplicon_OU4  .......... .......... .......... .......... .......... .......... .......... .......... .......... .......... .......... .......... ..........

ponAbe2       CAACAATGGT ATTTTTAATG ACAACATCAA ATGATCAACT GTGTAATCCA GTAATAAATT CAGAAATTTA GCCAAGCTTG GTGGCACGCA GCTGTATTCA ACTATTTCAA CTAATAGTTG GGGAAGCTGA
ponAbe3       .......... .......... .......... .......... .......... .......... .......... .......... .......... .......... .......... .......... ..........
Amplicon_OU4  .......... .......... .......... .......... .......... .......... .......... .......... .......... .......... .......... .......... ..........

ponAbe2       GGCAGAAGGA TCTCTTTAGC CTGAGGGTTC AAAGCTGTAA TGAGCTATGA TAGCATCCCT GCACTCCAGC CTGGGTGACA AAGCAAGACT CTGTCATAAA TAAATAAATA AAAGGAGGCT GGGTGTGGTG
ponAbe3       .......... .......... .......... .......... .......... .......... .......... .......... .......... .......... .......... .......... ..........
Amplicon_OU4  .......... .......... .......... .......... .......... .......... .......... .......... .......... .......... .......... .......... ..........

ponAbe2       GCTGAGTCAC CTGAGGTCAG GAGTTCAAGA CCAGCCTGGC CAACAAGGTG AAACCTCGTC TCTACTAAAA ATACAAAAAA ATTAGCCAGG CGTGGTGGTG CACACCTGTA TTGCCAGCTA CTCTGGAGGC
ponAbe3       .......... .......... .......... .......... .......... .......... .......... .......... .......... .......... .......... .......... ..........
Amplicon_OU4  .......... .......... .......... .......... .......... .......... .......... .......... .......... .......... .......... .......... ..........

ponAbe2       TGAGGCAGAA GAATTGCTTG AACCCGAGAG GCAAAGGTTG CAGTGAGCCG AGATCGCACC ACGGCACTCT GGCCTCGGTG ACAGAGCAAG ACTCCATCTC AAAGAAAAAA AAAAAGAAAA AGAAAAAGGG
ponAbe3       .......... .......... .......... .......... .......... .......... .......... .......... .......... .......... .......... .......... ..........
Amplicon_OU4  .......... .......... .......... .......... .......... .......... .......... .......... .......... .......... .......... .......... ..........
```

```
ponAbe2        AAAGGGGAAA GAAAAGGAAA CCTTGAATGT AGAACAGAGG TTCTCACACT TGAGTAAGCA TAAGAATCAC CTGGAGTGCT TATTAAACAC TGTAGGTTCA ATGACATTCC CTCAGAGATT CTGATTCAAT
ponAbe3        .......... .......... .......... .......... .......... .......... .......... .......... .......... .......... .......... .......... ..........
Amplicon_OU4   .......... .......... .......... .......... .......... .......... .......... .......... .......... .......... .......... .......... ..........

ponAbe2        TGGTCTGGGG ATCACCTGAC CTCCCCACCA CCATCACAAG GTCATCCTGA TACGAGTGGC CCAAAGACCA CACTTTAAGA CTGGCCTAAA CTCTAACATT TGCTATATCC TTCCTCTAAC TGGCCAAATC
ponAbe3        .......... .......... .......... .......... .......... .......... .......... .......... .......... .......... .......... .......... ..........
Amplicon_OU4   .......... .......... .......... .......... .......... .......... .......... .......... .......... .......... .......... .......... ..........

ponAbe2        GATAAAAACT CCAGGGCTCT CAGTTTTCAC CTCTCAAACA
ponAbe3        .......... .......... .......... ..........
Amplicon_OU4   .......... .......... .......... ..........
```

Binding sites of amplification primers are highlighted in yellow; *Alu*-like domain and SINE-R in green. Target site duplications are italicized and underlined.

Figure S4 The minimal polyA signal used in the *mneoM* cassette facilitates correct polyadenylation of neo cDNA

GAGTTCTTC**TGA**GG*GCGGCCGC***AATAAA**ATATC*TTTATT*TTCATTACATCT**GTGTGTTGGTTTTTTGTGTG**A*ATCGAT*

GAGTTCTTC**TGA**GG*GCGGCCGC***AATAAA**ATATCTTTATTTTCATTA*AAAAAAACAAAAAAAAAAAAAAAA*

3‘ RACE analysis to assess correct polyadenylation of the neomycin phosphotransferase gene using the minimal functional polyA signal (Levitt et al. 1989).

The minimal polyA signal (pGL3-derived) was tested downstream of an SV40 promoter-driven neomycin phosphotransferase cDNA. The stop codon is shown in red; the polyA signal and GU-rich tract are underlined. The polyA signal mediating premature polyadenylation of elements upstream of the reporter cassette is italicized and underlined.

Levitt N, Briggs D, Gil A, Proudfoot NJ. Definition of an efficient synthetic poly(A) site. Genes Dev. 1989; 3: 1019-1025.

Figure S5 Human SVA H8_43 *mneoM de novo* integrations.

#2D 5′-CTTAATAAATCACATCTGGAGAT//TG 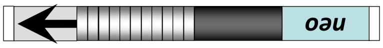 (A)$_n$ AATAAATCACATCTGCAT-3′

Pre-integration-site:

chr8:62,686,430

CTTTGTGCTT AATAAATCACATCTG CATCCACAGA
GAAACACGAA TTATTTAGTGTAGAC GTAGGTGTCT

#2C 5′-TTTAGAAGTTAAAAAAATTGAGAT//TG 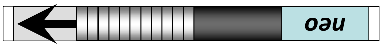 (A)$_n$ AGAAGTTAAAAAAATTTTC-3′

Pre-integration-site:

chr5:37,217,408

GTATCAATTT AGAAGTTAAAAAAATT TTCAAAGTGT
CATAGTTAAA TCTTCAATTTTTTTAA AAGTTTCACA

#1H 5′-CTTTAAAGTAATGATTTGAGAT//TG 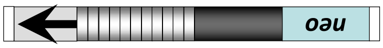 (A)$_n$ TAAGGTAATGATTTAAA-3′

Pre-integration-site:

chr20:50,562,112

AATTAATCTT TAAAGTAATGATTT AAAAAATACA
TTAATTAGAA ATTTCATTACTAAA TTTTTTATGT

The L1 endonuclease cleavage site on the bottom strand is indicated in blue. Extra G residues at the 5′-ends of the insertions are shown in green; target site duplications in red. Neo - neomycin-phosphotransferase gene.

Table S6 Sequences of oligonucleotides used in amplification and re-amplification of human and orangutan SVA elements.

Name	Sequence 5' -> 3'	observations
SVA_H8_43_F2	ACCTGACAACAAACCCTAAAATG	amplification from genome
SVA_H8_43_REV	TCGATGACTTCTATACTGCAACG	amplification from genome
H8_43_Kpn	GC<u>GGTACC</u>TATCGAAAGCTGATGAAATGCTC	re-amplification
H8_43_Nhe	AT<u>GCTAGC</u>GATCATTCTTGGGTGTTGGGATTTG	re-amplification
OU3_FW	GCAAATACGGTTGTAAACACAGG	amplification from genome
OU3_REV	GCATCCATTCAGTCAGTTAGGG	amplification from genome
OU3_Kpn	GC<u>GGTACC</u>ATGTATCAGCTGCTCTTGAG	re-amplification
OU3_Nhe	TT<u>GCTAGC</u>GATCATTCTTGGGTGTTTCTCG	re-amplification
OU4_FW	AACATAGGTAACATGGCACTGG	amplification from genome
OU4_REV	TGTTTGAGAGGTGAAAACTGAGAG	amplification from genome
OU4_Kpn	GC<u>GGTACC</u>CTTAGAATACAAGCTTTCTG	re-amplification
OU4_Nhe	TT<u>GCTAGC</u>GATCATTCTTGGGTGTTTCTCGGAGAG	re-amplification
OU6_FW	TGTAAAGAAAGAGACAGTGCTTGG	amplification from genome
OU6_REV	GGCTTATTGTAGTTATGCCTTTG	amplification from genome

Restriction enzyme recognition sites present in the re-amplification primers are underlined.
